# Off-Target Activation of CatSper Limits the Utility of a Novel Small Molecule Slo3 Inhibitor and its Derivatives in Human Sperm

**DOI:** 10.1021/acsptsci.6c00091

**Published:** 2026-08-31

**Authors:** Teresa Mittermair, Jens Münchow, Timo Strünker, Louisa Temme, Christoph Brenker

**Affiliations:** † Centre of Reproductive Medicine and Andrology, University Hospital Münster, 9185 University of Münster, Münster 48149, Germany; ‡ Institute of Pharmaceutical and Medicinal Chemistry, Pharmacampus, University of Münster, Münster 48149, Germany; § Institute of Pharmacy, University of Hamburg, Hamburg 20148, Germany

**Keywords:** human sperm, Slo3, CatSper, VU0546110

## Abstract

The sperm-specific K^+^ channel Slo3 is required for sperm function and fertilization in mice and humans. Due to the lack of selective pharmacological tools, its molecular and cellular physiology in human sperm has remained only ill-defined, though. The compound VU0546110 (**1**) was recently introduced as the first selective Slo3 inhibitor and is now being used to study the channel’s role in human sperm. Whether the compound may have off-target actions in sperm is however unknown. Here, using kinetic Ca^2+^ fluorimetry, patch-clamp recordings, and sperm from infertile men with a homozygous deletion of the *CATSPER*2 gene as experimental model, we show that VU0546110 simultaneously inhibits Slo3 and activates the sperm-specific Ca^2+^ channel CatSper. This demonstrates that the utility of the drug is limited: it is difficult to decipher if its action on human sperm is due to inhibition of Slo3, activation of CatSper, or a combination of both. We also introduce derivatives of VU0546110 that show a reduced potency and efficacy for CatSper activation, while preserving the inhibitory action on Slo3.

## Graphical abstract

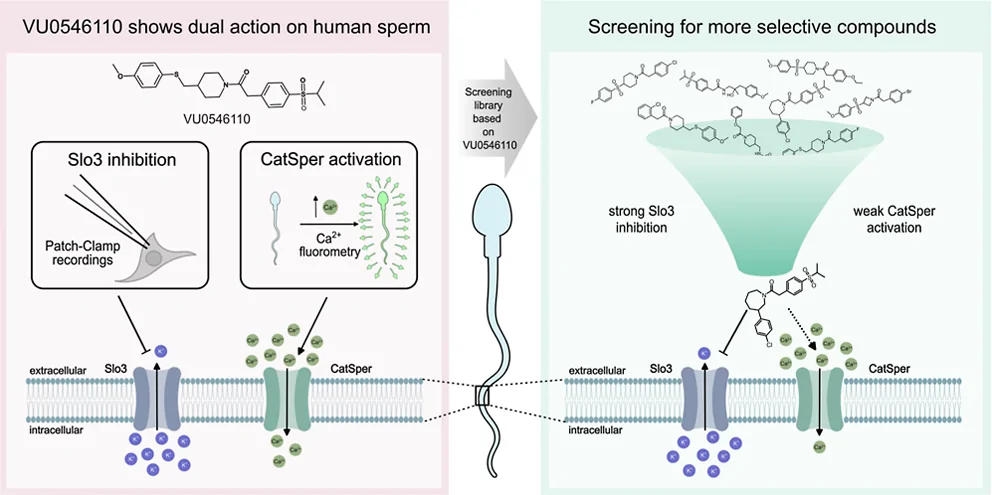

The function of human sperm is controlled by changes in the intracellular Ca^2+^ concentration ([Ca^2+^]_i_), intracellular pH (pH_i_), and membrane potential (*V*_m_), mediated by a network of mostly sperm-specific ion channels and transporters.^1–8^ Prime examples are CatSper (Cation channel of Sperm) and Slo3 channels that are localized in the flagellum. CatSper is the principal Ca^2+^ channel in mouse and human sperm and essential for sperm function and fertilization.^8–17^ Sperm lacking functional CatSper are unable to switch to so-called hyperactivated motility, i.e., a powerful flagellar beat characterized by high asymmetry and low frequency, which is required to penetrate the egg coat. Therefore, loss of CatSper function results in male infertility.^16–19^ In humans, CatSper serves as a polymodal chemosensor that is modulated by a wide range of physiological ligands and synthetic compounds.^20–34^ Slo3 is the principal K^+^ channel in mouse^1,35–38^ and human sperm^39,40^ and is also essential for sperm function and fertilization. Male mice that lack Slo3 are infertile.^36,37^ In humans, loss of Slo3 function is also associated with male infertility. However, in contrast to CatSper, the precise role of Slo3 in human sperm has not yet been resolved. For example, human Slo3 is activated by depolarization of the *V*_m_, an increase of pH_i_, and, most strongly, by an increase of [Ca^2+^]_i_.^39,41^ Thereby, Slo3 regulates the V_m_ of human sperm in a Ca^2+^-dependent manner;^39,42^ and CatSper-mediated Ca^2+^ signaling involves a dynamic interplay between CatSper and Slo3.^43^ Elucidating the molecular mechanistic role of Slo3 in this interplay and, thereby, in the regulation of human sperm function would require studying Ca^2+^ signaling in sperm from men lacking functional Slo3.^44^ Yet, men with loss of Slo3 function are scarce and, thus, not available as experimental models. Thus far, only three infertile men with pathogenic variants resulting in loss of Slo3 function have been described.^44,45^

Therefore, to delineate the role of Slo3 in human sperm, selective inhibitors of the channel are required. Compounds that selectively inhibit human Slo3 have, however, been lacking. A key problem for compound development is that, in human sperm, the pharmacological profile of Slo3 largely overlaps with that of CatSper. For instance, human Slo3 is inhibited by steroids that activate CatSper.^39,46^ Human Slo3 is also inhibited by the CatSper inhibitors NNC55-0396,^20,21^ Mibefradil,^20,47,48^ RU1968,^26^ TS150,^49^ MDL12330A,^50^ and the triazolopyridazine 7a.^51^ *Vice versa*, human CatSper is inhibited by the K^+^ channel inhibitors quinidine and clofilium,^48^ which also inhibit Slo3.^39^ The absence of tool compounds that discriminate between Slo3 and CatSper has hindered exploring Slo3′s physiological function in humans.

Only recently, this long-standing issue was seemingly solved as the discovery of the first selective Slo3 inhibitor, VU0546110 (**1**) (Figure 1A), was reported.^40^ Indeed, VU0546110 (**1**) is highly selective for Slo3 over Slo1, and the compound completely inhibited K^+^ currents in human sperm. This finding confirmed that Slo3 ^39^ but not Slo1 ^42^ is the principal K^+^ channel in human sperm—as demonstrated before by combining protein-mass spectrometric, pharmacological, and single-channel conductance analyses in human sperm, as well as by comparing the properties of heterologous human Slo3 and human Slo1 with that of K^+^ currents in human sperm.^39^ Moreover, VU0546110 (**1**) affected human sperm hyperpolarization, acrosome reaction, and hyperactivated motility,^40^ which led to the conclusion that these functions of human sperm are regulated by Slo3. The problem is, however, that it was not investigated whether VU0546110 (**1**) may also act on CatSper.

**Figure 1:**
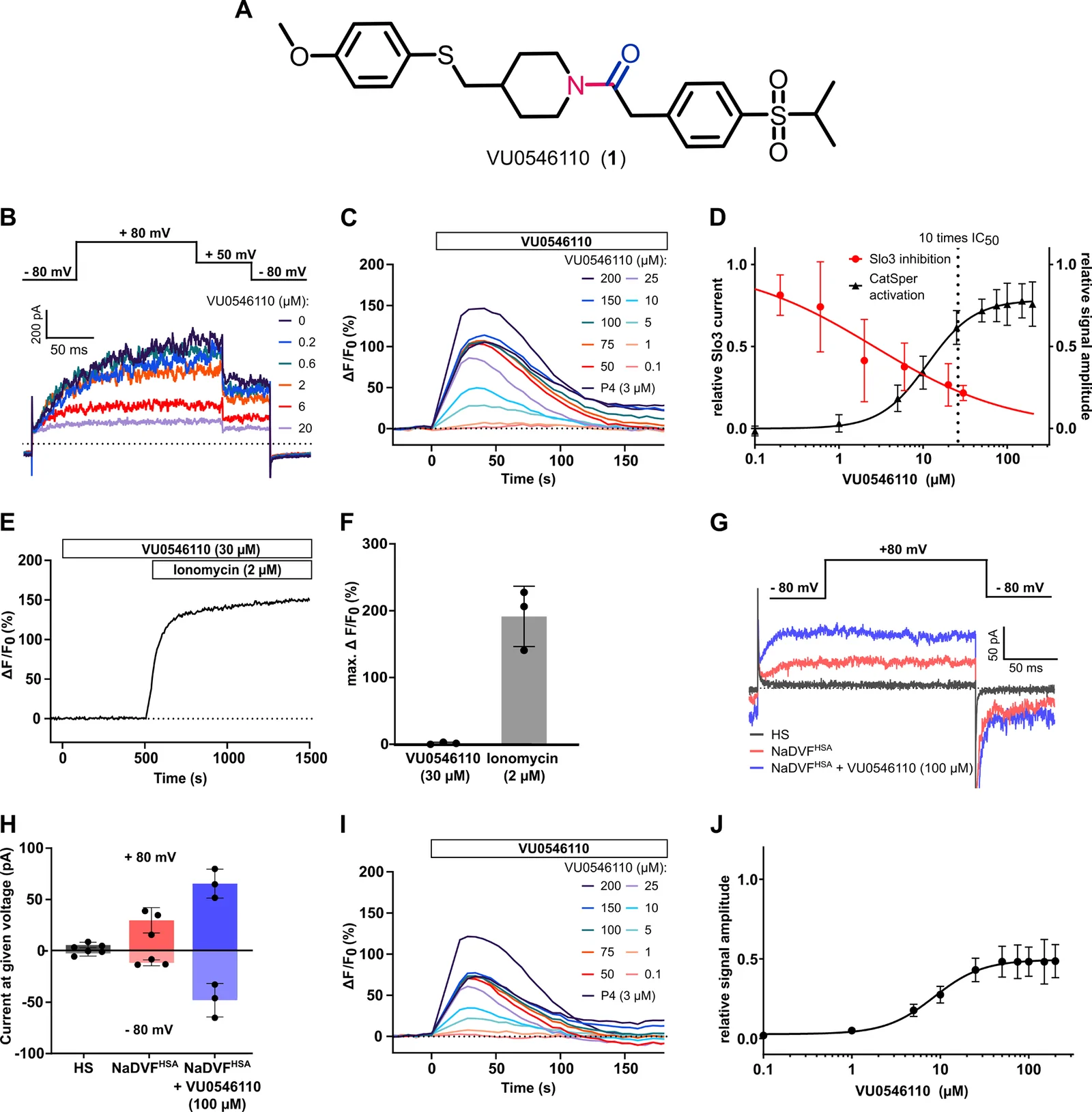
Action of VU0546110 on heterologous human Slo3 and CatSper in human sperm. (A) Structural formula of VU0546110 (**1**). The central amide moiety is marked in red and blue. (B) Membrane currents recorded in the whole-cell configuration from CHO cells coexpressing human Slo3 and LRRC52 before and after perfusion with different concentrations of VU0546110 (**1**). (C) Representative Ca^2+^ signals in human sperm evoked by different concentrations of VU0546110 (**1**) and progesterone (3 μM). ΔF/F_0_ (%) indicates the percentage change in fluorescence (Δ*F*) with respect to the mean basal fluorescence (*F*_0_) before the application of VU0546110 (**1**) or progesterone (3 μM). (D) Slo3 over CatSper selectivity window of VU0546110. Red: Slo3-current amplitudes (mean ± SD) at +80 mV in the presence of different concentrations of VU0546110 (**1**) relative to that under control conditions (set to 1) (*n* ≥ 4). The continuous line represents a fit of the Hill equation yielding the dose–response relationship. Black: dose–response relation of maximal Ca^2+^-signal amplitudes evoked by VU0546110 (**1**) in human sperm incubated in HTF, relative to that evoked by progesterone (3 μM) (set to 1). (E) Ca^2+^ signals in *CATSPER2* ^–/–^ sperm evoked by VU0546110 (**1**, 30 μM) and subsequent stimulation by ionomycin (2 μM). (F) Maximal signal amplitude evoked by VU0546110 (**1**, 30 μM) and ionomycin (2 μM) in *CATSPER2* ^–/–^ sperm (mean ± SD, *n* = 3). (G) Representative CatSper-mediated membrane currents recorded from a human sperm cell in the whole-cell configuration in extracellular solution containing Ca^2+^ and Mg^2+^ (HS), in divalent-free Na^+^-based bath solution (NaDVF^HSA^) containing HSA (15 μM), and after perfusion with NaDVF^HSA^ containing VU0546110 (**1**, 100 μM). (H) Mean (±SD) control currents in HS and CatSper currents in NaDVF^HSA^ in the absence and presence of VU0546110 (**1**, 100 μM) (*n* = 3). (I) Representative Ca^2+^ signals in human sperm evoked by different concentrations of VU0546110 (**1**) and progesterone (3 μM) incubated in high-potassium HTF (K-HTF). (J) Dose–response relation of maximal signal amplitudes evoked by VU0546110 (**1**) in human sperm incubated in K-HTF relative to that evoked by progesterone (3 μM) (set to 1) (*n* = 4).

Here, we studied the action of VU0546110 (**1**) on CatSper in human sperm. We show that VU0546110 (**1**) simultaneously inhibits Slo3 and activates CatSper. Thereby, the compound stimulates a rapid increase in flagellar Ca^2+^ levels, resembling the action of physiological CatSper agonists like progesterone. This off-target activation of CatSper limits the utility of VU0546110. Therefore, we also investigated the action of 30 commercially available VU0546110 (**1**) derivatives on human Slo3 and CatSper, and we designed and synthesized two compounds that combine their scaffolds. This approach identified compounds with higher selectivity for Slo3 over CatSper than VU0546110 (**1**) that seem to be better suited, but are still far from ideal, as tools to elucidate the role of Slo3 in human sperm.

## Results

### VU0546110 Inhibits Slo3 but also Activates CatSper in Human Sperm

First, we investigated the action of VU0546110 (**1**) (Figure 1A) on human Slo3 transiently coexpressed with its auxiliary subunit LRRC52 in CHO cells. We recorded Slo3-mediated membrane currents before and after perfusion with different concentrations of the compound. In line with the results by Lyon et al.,^40^ VU0546110 (**1**) inhibited the Slo3 currents with an IC_50_ of 2.6 ± 0.4 μM (standard error of the fit, SE) (Figure 1B,D). Next, we investigated the action of VU0546110 (**1**) on CatSper in human sperm. We challenged human sperm with progesterone (control) or the compound and monitored the changes in the intracellular Ca^2+^ concentration ([Ca^2+^]_i_) using the fluorescent Ca^2+^ indicator Fluo-4. Similar to progesterone, VU0546110 (**1**) evoked a transient [Ca^2+^]_i_ increase that peaked within 40 s after stimulation (Figure 1C). Then, [Ca^2+^]_i_ decreased again and settled near resting levels within about 150 s. The amplitude of the VU0546110-induced Ca^2+^ signal increased in a dose-dependent fashion with an EC_50_ of 12.6 ± 1.2 μM (standard deviation, SD, *n* = 4) (Figure 1D). Of note, the experiments were performed in HTF buffer containing about 0.1 mg/mL (i.e., 1.5 μM) human serum albumin (HSA), as it enhanced the amplitude of VU0546110-induced Ca^2+^ signals (Figure S1); the signal waveform was, however, similar in the presence and absence of HSA. In CatSper-deficient sperm from men lacking the *CATSPER*2 gene (*CATSPER*2^–/–^),^17,31,33,52^ the Ca^2+^ signal evoked by VU0546110 (**1**) was absent (Figure 1E,F), demonstrating that it rests on Ca^2+^ influx through CatSper. The [Ca^2+^]_i_ increase evoked by the Ca^2+^-ionophore ionomycin was preserved in *CATSPER*2^–/–^ sperm (Figure 1E,F), serving as a control scrutinizing our experimental conditions. We also investigated whether VU0546110 features a dual agonistic-inhibitory action on CatSper, as observed for CatSper inhibitors such as RU1968 and derivatives,^26,49^ sertraline,^32^ and THC.^58^ In the presence of 30 μM of the compound, the amplitude of the progesterone-evoked Ca^2+^ signal was only slightly reduced, i.e. by 23.3 ± 2.3% (mean ± SD, *n* = 4, Figure S2), indicating that VU0546110 is a genuine CatSper agonist. To support this conclusion by an independent technique, we also studied the action of VU0546110 (**1**) on CatSper-mediated membrane currents in human sperm. The compound increased the CatSper currents at +80 mV and −80 mV by 2.5 ± 0.7- and 4.2 ± 1-fold, respectively (mean ± SD, *n* = 3), also demonstrating that it directly activates the channel (Figure 1G,H). Yet, the activation of CatSper by VU0546110 (**1**) required HSA in the extracellular solution (compare Figures 1 G,H and S3). We did not further investigate the role of HSA in the action of the compound on CatSper.

The dose–response curves for VU0546110 to stimulate Ca^2+^ influx via CatSper and to inhibit Slo3 currents largely overlap (Figure 1D). Therefore, we tested whether the VU0546110-evoked influx of Ca^2+^ through CatSper may be indirectly enhanced by depolarization caused by the inhibition of Slo3; CatSper, like Slo3, is activated by depolarization. We studied the action of the compound on sperm bathed in buffer containing 150 mM K^+^ (K-HTF), which depolarizes *V*_m_ ^39,43,53^ and, thereby, mimics inhibition of Slo3. In K-HTF, VU0546110 (**1**) also evoked a transient [Ca^2+^]_i_ increase with an EC_50_ of 8.5 ± 1.8 μM (*n* = 4) (Figure 1I,J), i.e., similar to that in control HTF.

Yet, an indirect depolarization-induced enhancement of Ca^2+^-influx via CatSper through inhibition of Slo3 in control HTF should be reflected by a decrease in the Ca^2+^-signal amplitude in K-HTF versus control HTF. Indeed, the maximal signal amplitude—relative to that evoked by progesterone (set to 1)—was reduced in K-HTF (0.49 ± 0.09, *n* = 4) versus control HTF (0.67 ± 0.12, *n* = 4), whereas the progesterone-evoked Ca^2+^ signals were similar in amplitude in both conditions (Figure S4). This demonstrates that VU0546110-induced Ca^2+^ signals in human sperm rest predominantly on the direct activation of CatSper, but seem to be slightly enhanced by depolarization caused by inhibition of Slo3.

In summary, under physiological conditions, VU0546110 (**1**) is a dual-target drug that simultaneously acts on human CatSper and Slo3. Therefore, the drug is unsuited to elucidate the role of Slo3 in human sperm: it is difficult if not impossible to decipher if its action on the function of human sperm is due to inhibition of Slo3, activation of CatSper, or a combination of both.

### Identification of Slo3 Inhibitors with Improved Selectivity

We set out to identify compounds that inhibit human Slo3 at least as potently as VU0546110 (**1**), but feature a reduced potency and/or efficacy to activate CatSper. We selected 30 commercially available derivatives of VU0546110 (**1**) with a Jaccard-Tanimoto similarity coefficient of ≥0.7, calculated using the RDKit fingerprint with default settings provided by the vendor. The Jaccard-Tanimoto similarity coefficient quantifies molecular similarity by comparing the number of shared features between two molecules to the total number of distinct features found across both molecules.^59^ The selected compounds **2**–**31** only share the amide moiety with VU0546110 (**1**) (Figure 2). Of note, as **5**, **6**, **24**, **25**, **26**, **27**, **28**, and **29** were obtained as racemic mixtures, it cannot be excluded that the respective enantiomers may feature different pharmacologic properties. Nevertheless, we first tested their action on CatSper. To this end, we challenged human sperm in K-HTF with the compounds (30 μM), monitored the resulting changes in [Ca^2+^]_i_, and grouped the compounds according to the amplitude of the Ca^2+^ responses in Classes A, B, and C (Figure 3). In the following, to ease readability, we refer to the compounds as AX, BX, or CX. The eight compounds grouped in Class A, e.g., A2 and A4 (Figure 3A), evoked Ca^2+^ signals with a similar amplitude as VU0546110 (**1**), suggesting that they activate CatSper with similar potency and/or efficacy. The 16 compounds grouped in Class B, e.g., B8 and B9, evoked Ca^2+^ signals with smaller amplitudes compared to VU0546110 (**1**) (Figure 3B). The Ca^2+^ signals evoked by the Class A and B compounds were largely abolished in *CATSPER*2^–/–^ sperm, demonstrating that they rest on Ca^2+^ influx through CatSper (Figure 3D). Six compounds did not affect [Ca^2+^]_i_, e.g., C1 and C6 (Figure 3C). Together, these results suggest that the Class B compounds might activate CatSper with a reduced potency/efficacy compared to VU0546110 (**1**), whereas compounds in Class C might not activate CatSper at all. We also tested a potential inhibitory action on CatSper. At the test concentration of 30 μM, most compounds did not affect the amplitude of progesterone-induced Ca^2+^ signals. Like VU0546110, some compounds slightly reduced the signal amplitude – by only ≤25%, though (Figure S2). This indicates that none of the compounds acts as a genuine CatSper inhibitor.

**Figure 2:**
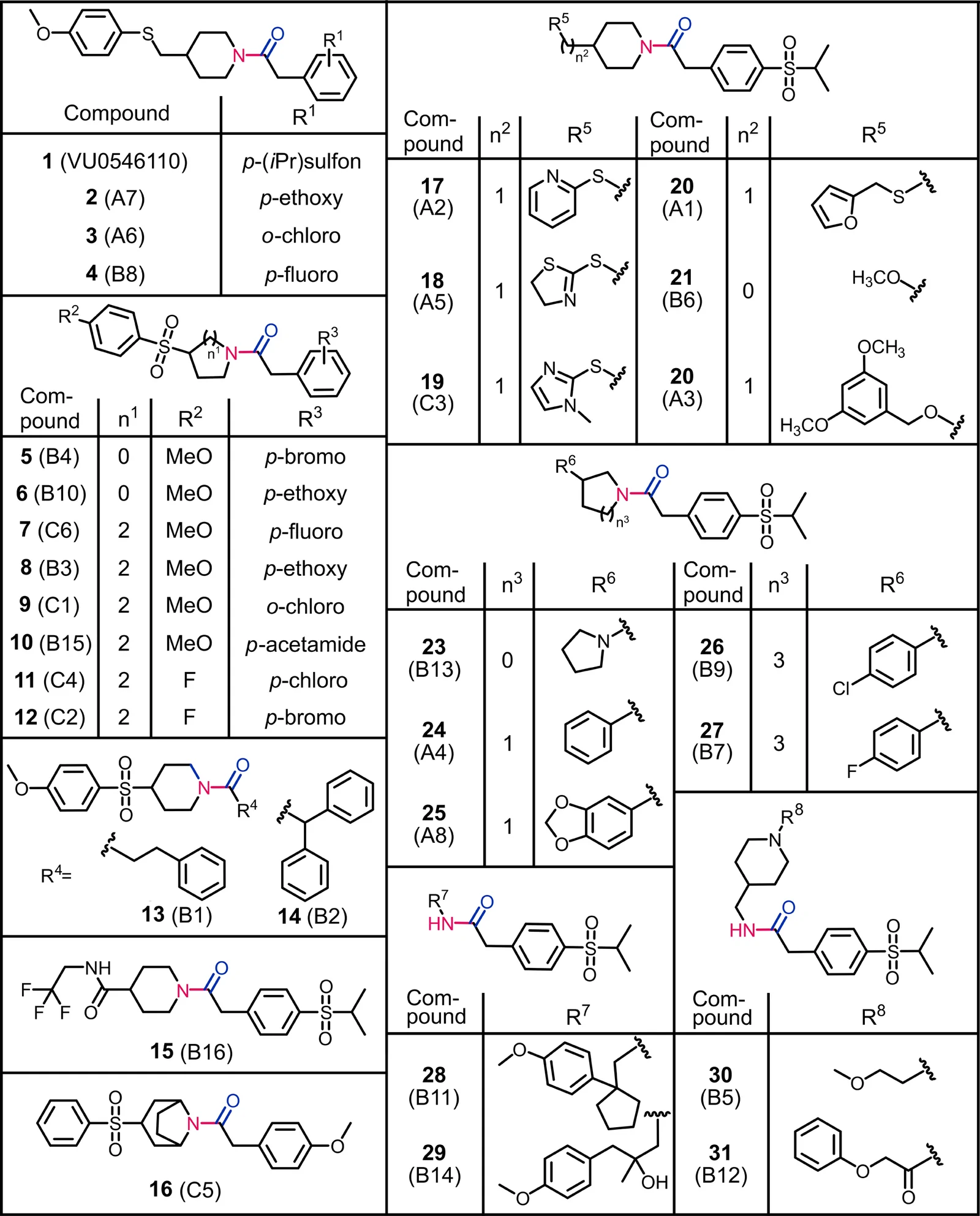
Set of VU0546110-derived compounds selected to screen for selective Slo3 inhibition. The central amide moiety of the compounds is marked in red and blue. Number in brackets indicate the grouping and numbering by biological activity (for details see Figure 3). *n* ^x^ gives the number of C atoms and *R* ^x^ the moiety attached.

**Figure 3:**
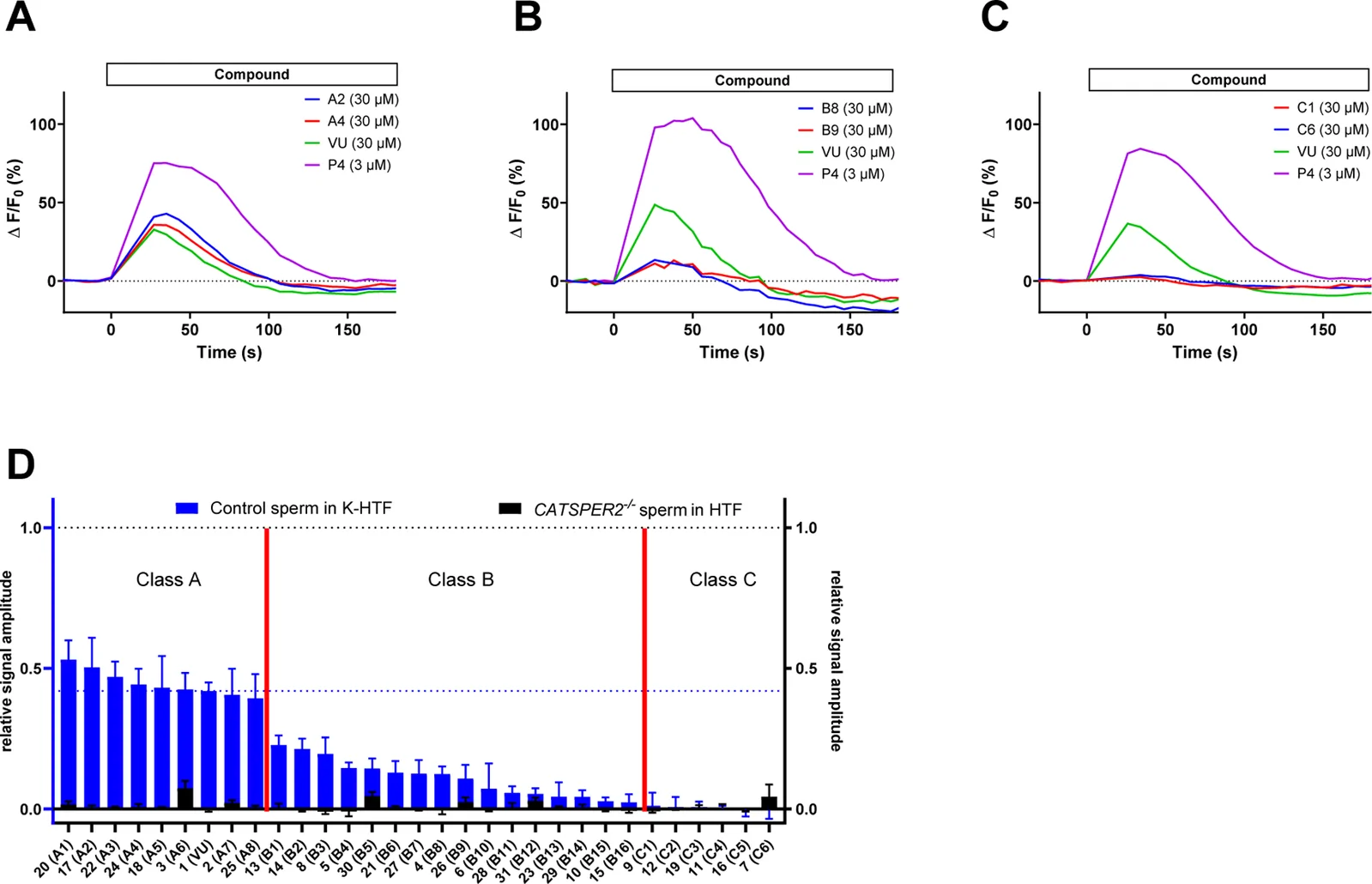
Action of VU0546110-derived compounds on CatSper in human sperm. (A–C) Representative Ca^2+^ signals in human sperm evoked by selected compounds (30 μM), VU0546110 (30 μM), and progesterone (3 μM) in K-HTF. Δ*F*/*F*_0_ (%) indicates the percentage change in fluorescence (Δ*F*) with respect to the mean basal fluorescence (*F*_0_) before the application of VU0546110 (**1**) or progesterone (3 μM). (D) Blue, left *Y*-axis: maximal (±SD) signal amplitude of Ca^2+^ signals in human sperm evoked by compounds (30 μM) in K-HTF, relative to that evoked by progesterone (set to 1). Black, right *Y*-axis: maximal (±SD) amplitude of Ca^2+^ signals in *CATSPER*2^–/–^ sperm evoked by compounds (30 μM), relative to that evoked by ionomycin (2 μM) (set to 1) (*n* = 3). Number in brackets indicate the numbering and grouping of the compounds according to the amplitude of the Ca^2+^ responses in Classes A, B and C. See also the explanation in the text.

We next investigated the agonistic action of the Class C and B compounds in more detail. To gauge the inhibitory action of the compounds on Slo3, we studied the compound-evoked Ca^2+^ signals in control HTF and compared the signal amplitudes to those observed in K-HTF. In control HTF, but not in K-HTF, inhibition of Slo3 leads to membrane depolarisation, which indirectly enhances Ca^2+^-influx via CatSper. We assumed that with increasing potency and/or efficacy of a compound to inhibit Slo3, the decrease in signal amplitude in K-HTF versus control HTF becomes more pronounced. The amplitudes of the VU0546110-evoked signals served as the reference (Figure 4G, red data points and line). C3–C6 did neither affect [Ca^2+^]_i_ in K-HTF nor in control HTF (Figure 4G, blue data points), and for several Class B compounds (black data points in Figure 4G), e.g., B10-B16, and B5 (Figure 4B), Ca^2+^ signals were similar in HTF versus K-HTF. This suggests that these compounds feature a much lower potency and/or efficacy as VU0546110 (**1**) to inhibit Slo3 or do not inhibit the channel at all. By contrast, C1 and C2 evoked small, but sizable Ca^2+^ signals in control HTF but not in K-HTF (Figure 4E,G), suggesting that the drugs are weak inhibitors of Slo3. Compounds B1-B4 and B7-B9 also showed differences in signal amplitudes in HTF versus K-HTF; for B2, B8, and B9, this difference was even more pronounced than for VU0546110 (**1**), suggesting that these compounds inhibit Slo3 with similar or even higher potency and/or efficacy.

**Figure 4:**
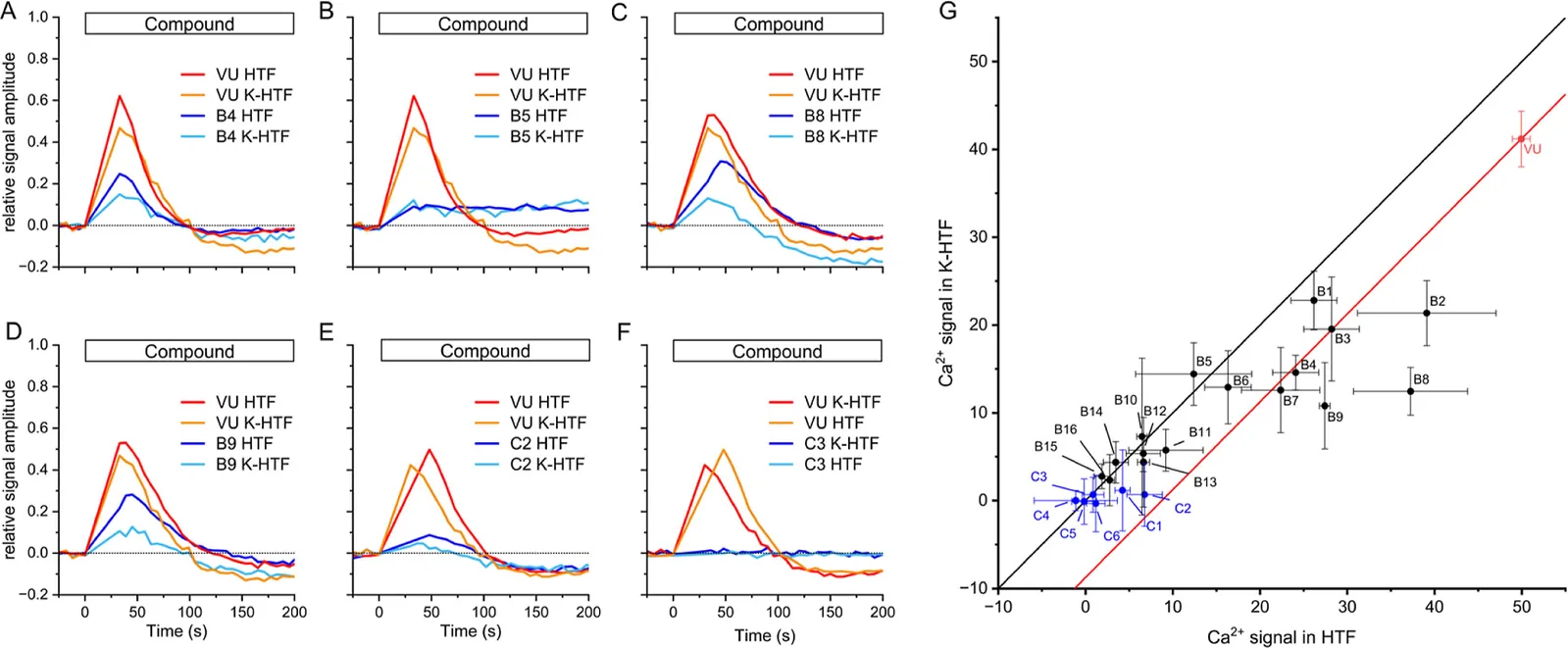
Ca^2+^ signals evoked by Class B and Class C compounds in K-HTF versus HTF. Representative Ca^2+^ signals in human sperm evoked by selected compounds (30 μM) and VU0546110 (30 μM) in HTF and K-HTF. To increase comparability between experiments, the Ca^2+^ signal amplitude was normalized to the signal amplitude evoked by progesterone in HTF and K-HTF (set to 1). (G) Maximal amplitude of compound-evoked Ca^2+^ signals (at 30 μM) normalized to the signal amplitude evoked by progesterone (3 μM) in K-HTF plotted against those in HTF (*n* ≥ 3). Compounds of Class C (30 μM) are shown in blue and VU0546110 (30 μM) is shown in red. Compounds that cluster on the black line show similar signal amplitudes in HTF and K-HTF, compounds that cluster on the red line show a similar difference in signal amplitudes in HTF versus K-HTF as VU0546110 (at 30 μM).

Indeed, barring a few exceptions (e.g., B2 and B4), the difference in Ca^2+^-signal amplitudes was predictive for the inhibitory action on Slo3. For example, at 30 μM, C3 did not affect Slo3 currents and C1, C2–C6, and B3 decreased the current amplitude only by ≤ 60%, confirming that the compounds are less potent and/or efficacious than VU0546110 (**1**) (Slo3 inhibition at 30 μM: 79 ± 5%) (Figure 5A,B). By contrast, B8 and B9 almost completely inhibited Slo3 currents. Thus, comparing compound-evoked Ca^2+^ signals in control HTF versus K-HTF represents a time-effective approach to screen for Slo3 inhibitors that are selective for Slo3 over CatSper, minimizing laborious electrophysiological experiments.

**Figure 5:**
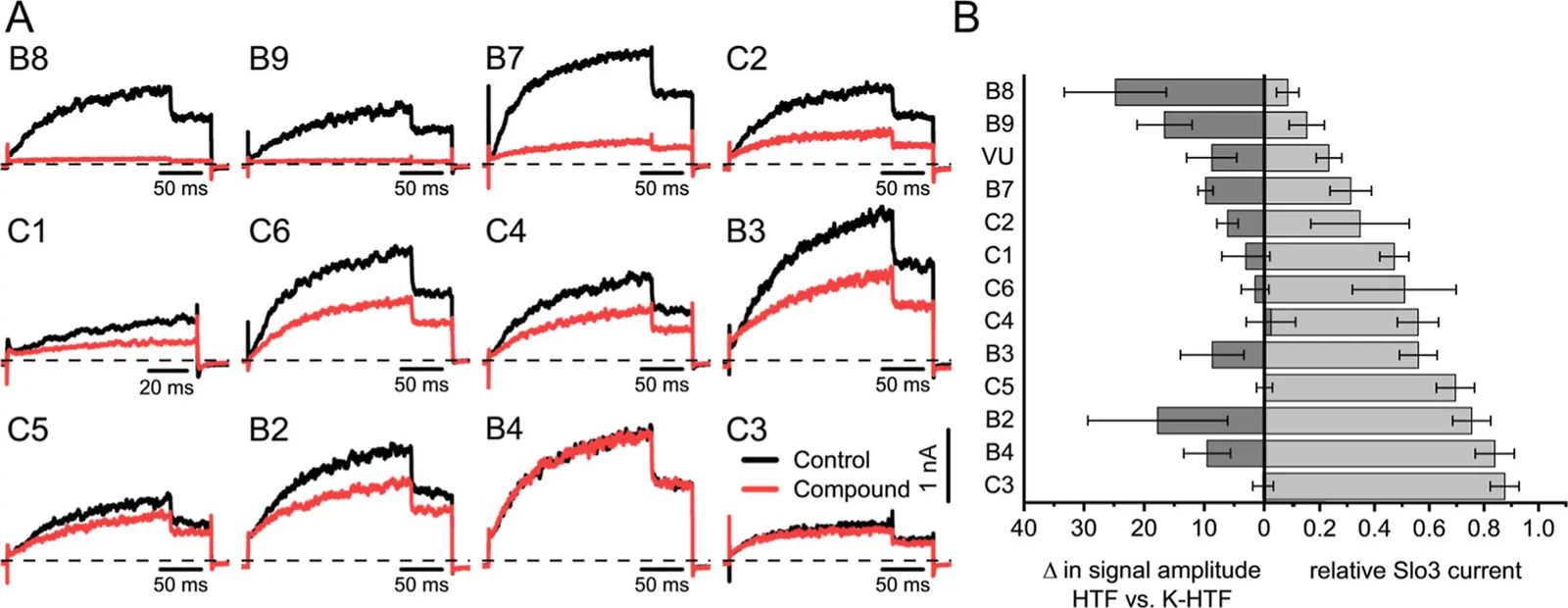
Action of Class B and C compounds on human Slo3. (A) Membrane currents recorded in the whole-cell configuration from CHO cells coexpressing human Slo3 and LRRC52 before (black) and after (red) perfusion with 30 μM of the indicated compound. (B) Difference (Δ) in the maximal Ca^2+^-signal amplitude in HTF vs K-HTF in comparison to Slo3-mediated currents (mean ± SD, *n* ≥ 3) in the presence of 30 μM of the respective compound relative to that recorded in its absence (set to 1).

To scrutinize this notion, we investigated the action of the most promising compounds, B8 and B9, on Slo3 and CatSper in more detail (Figure 6). Both drugs inhibited Slo3 currents in a dose-dependent fashion with IC_50_ values similar to that of VU0546110 (**1**), i.e., 4.8 ± 0.9 μM (B8) and 3.8 ± 0.3 μM (B9) (*n* ≥ 3, standard error of the fit, SE, Figure 6A–D). B8 evoked Ca^2+^ signals with similar potency (EC_50_ = 14.4 ± 2.4 μM, *n* = 3) (Figure 6E,F) as VU0546110, whereas the EC_50_ of B9 was increased (28.0 ± 12.2 μM, *n* = 5) (Figure 6G,H). However, B8 and B9 activated CatSper less, efficaciously than VU0546110: The maximal B8- and B9-induced signal amplitude was 0.37 ± 0.02 (*n* = 3, Figure 6E,F) and 0.30 ± 0.05 (*n* = 3, Figure 6G,H), respectively, relative to that evoked by progesterone (set to 1) (VU0546110 = 0.67 ± 0.12, *n* = 4).

This demonstrates that, even though B8, B9, and VU0546110 inhibit Slo3 with similar potency, B8 (**4**) and particularly B9 (**26**) are more selective for Slo3 over CatSper than VU0546110 (**1**).

**Figure 6:**
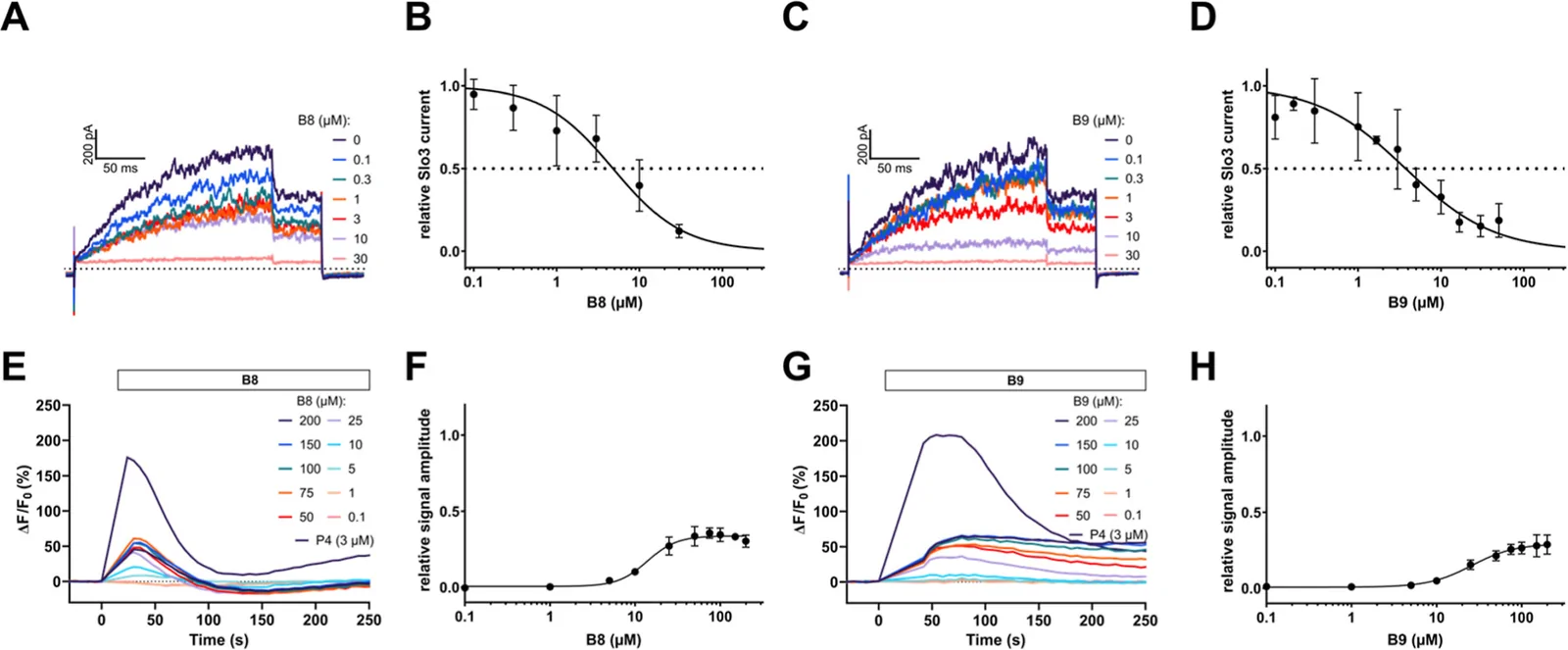
Action of compounds B8 and B9 on heterologous human Slo3 and on CatSper in human sperm. (A,C) Membrane currents recorded in the whole-cell configuration from CHO cells coexpressing human Slo3 and LRRC52 before and after perfusion with different concentrations of B8 and B9. (B,D) Current amplitudes (mean ± SD) at +80 mV in the presence of different concentrations of B8 and B9 relative to that under control conditions (set to 1) (*n* ≥ 3). Continuous lines represent fits of the Hill equation yielding the dose–response relationship. (E,G) Representative Ca^2+^ signals in human sperm evoked by different concentrations of B8, B9, and progesterone (3 μM) in HTF. Δ*F*/*F*_0_ (%) indicates the percentage change in fluorescence (Δ*F*) with respect to the mean basal fluorescence (*F*_0_) before the application of B8, B9 or progesterone (3 μM). (F,H) Dose–response relation of maximal signal amplitudes evoked by B8 and B9 in human sperm incubated in HTF relative to that evoked by progesterone (set to 1) (*n* = 5).

### Synthesis and Characterization of B8 and B9 Derivatives

We designed and synthesized new compounds aiming at even further improving their properties. The common structural feature of the tested compounds (**2**–**31**) and VU0546110 (**1**) is the central amide moiety. This divides the molecules in a carboxylic acid-derived moiety and a moiety descending from an amine. B9 (**26**) and B8 (**4**) share the acid and amine substructures with VU0546110 (**1**), respectively (Figure 7A). Each substructure might have beneficial effects on the selectivity of these compounds for Slo3 over CatSper. Therefore, we tested if combining the acid substructure of B8 (**4**) with the amine substructure of B9 (**26**) increases the selectivity further (Figure 7A). Hence, we designed TL6-49 (**32**) in which the *p*-isopropyl sulfonyl moiety of VU0546110 (**1**) is replaced by an F atom and the whole amine part of VU0546110 (**1**) is replaced by 3-(*p*-chlorophenyl)-azepane. To investigate also the effect of the *p*-chlorophenyl moiety of B9 (**26**), we envisaged the synthesis of TL6-47 (**33**), in which nonsubstituted azepane is used as the amine building block. TL6-47 (**33**) and TL6-49 (**32**) were synthesized in a condensation reaction of racemic azepane **35** or azepane **34**, respectively, with 4-fluorophenylacetyl chloride **36** in yields of 88% and 21%, respectively (Figure 7B). Of note, we did not separate the TL6-49 (**32**) enantiomers, but used the racemic mixture for our experiments. Thus, we cannot exclude that the two TL6-49 (**32**) enantiomers might feature different pharmacological properties. First, we studied the action of the compounds on Slo3. TL6-47 (**33**) did not inhibit Slo3 (Figure 8A), whereas TL6-49 (**32**) inhibited the channel with an IC_50_ of 1.6 ± 0.5 μM (standard error of the fit, SE, Figure 8B,C). These results indicate that the *p*-chloro aryl moiety is essential for inhibiting Slo3. Next, we studied the action of TL6-49 on CatSper. In human sperm, the compound evoked Ca^2+^ signals with an EC_50_ of 5.8 ± 0.8 μM (*n* = 4), indicating that TL6-49 (**32**) activates CatSper more potently than VU0546110 (**1**) (Figure 8D,E). The maximal relative signal amplitude was 0.43 ± 0.05 (*n* = 4), suggesting a reduced efficacy compared to VU0546110 (**1**).

**Figure 7:**
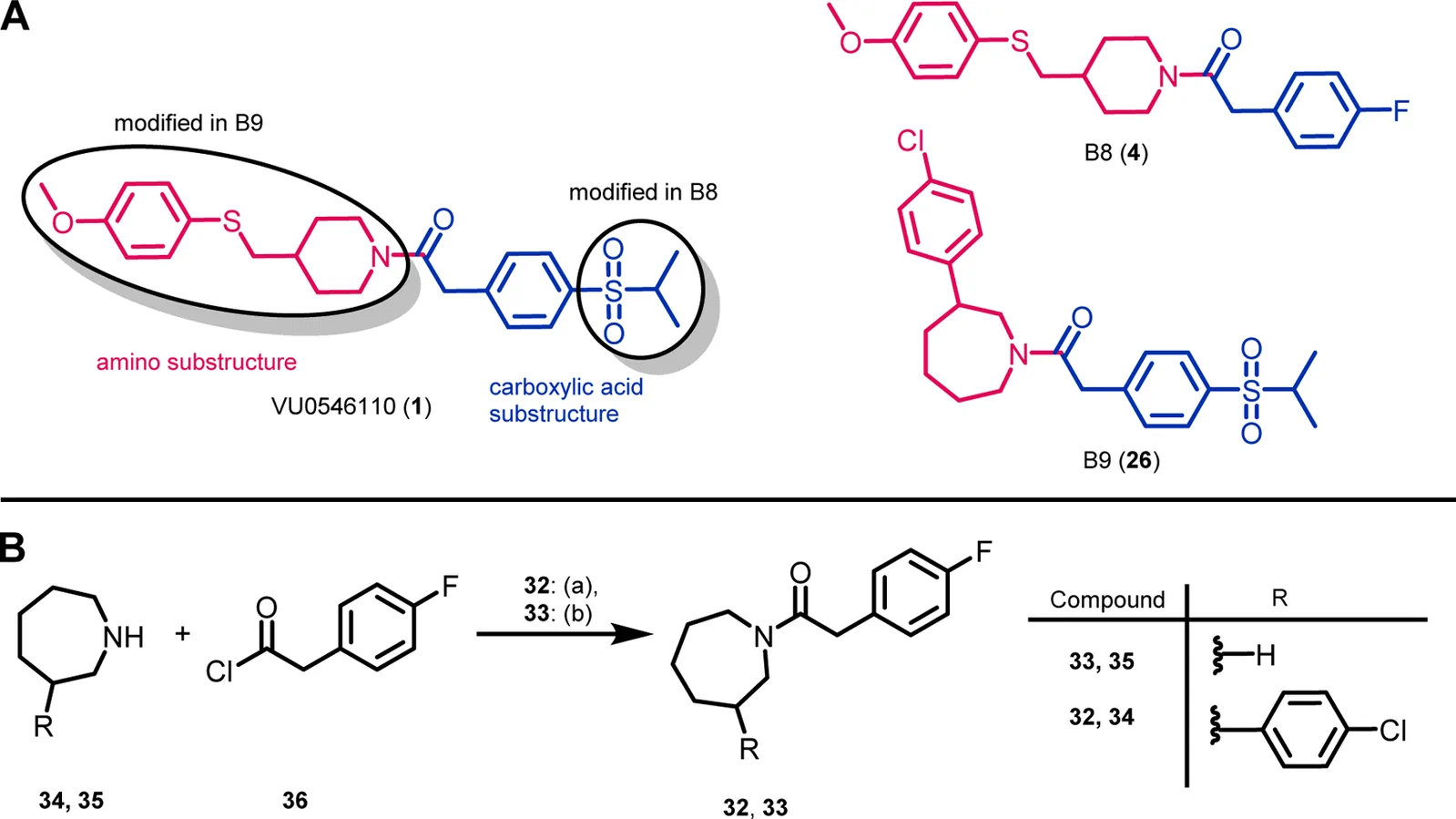
Structural analysis of VU0546110 (**1**), B8 (**4**), and B9 (**26**) as well as the synthesis of amides **32** and **33**. (A) Structural alterations of B8 (**4**) and B9 (**26**) compared to VU0546110 (**1**). (B) Condensation of azepane **34** or **35** with 4-fluorophenylacetyl chloride (**36**) leads to amides **32** (TL6-49) and **33** (TL6-47), respectively. (a) DMAP, CH_2_Cl_2_, 0 °C → room temperature, 80 min, 21%. (b) DMAP, CH_2_Cl_2_, 0 °C → room temperature, 1 h, 88%.

**Figure 8:**
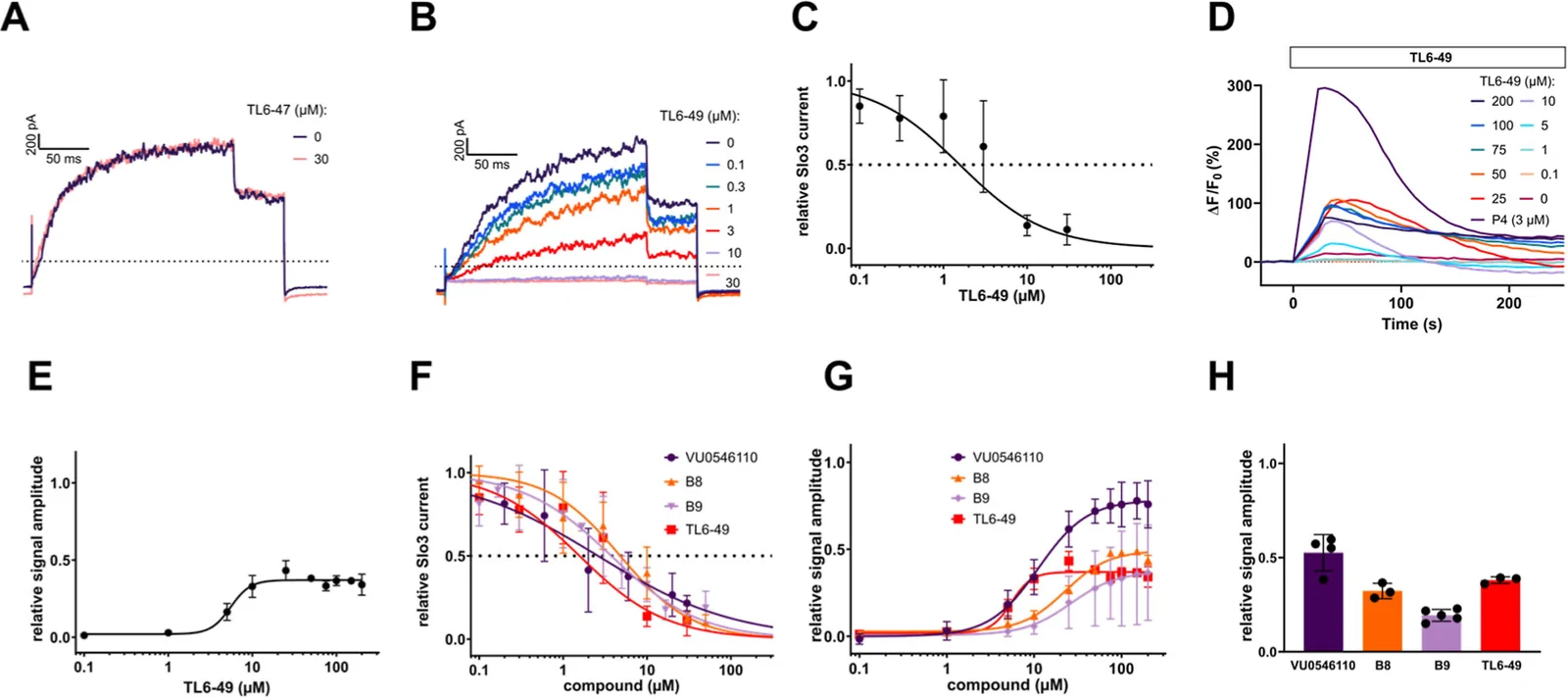
Action of TL6-47 and TL6-49 on heterologous human Slo3 and CatSper in human sperm. (A,B) Membrane currents recorded in the whole-cell configuration from CHO cells coexpressing human Slo3 and LRRC52 before and after perfusion with different concentrations of TL6-47 and TL6-49. (C) Current amplitudes (mean ± SD) at +80 mV in the presence of different concentrations of TL6-49 relative to that under control conditions (set to 1) (*n* ≥ 3). The continuous line represents a fit of the Hill equation yielding the dose–response relationship. (D) Representative Ca^2+^ signals in human sperm evoked by different concentrations of TL6-49 and progesterone (3 μM) in HTF. Δ*F*/*F*_0_ (%) indicates the percentage change in fluorescence (Δ*F*) with respect to the mean basal fluorescence (*F*_0_) before the application of TL6-49 and progesterone (3 μM). (E) Dose–response relation of maximal signal amplitudes evoked by TL6-49 in human sperm incubated in HTF relative to that evoked by progesterone (3 μM) (set to 1). (F) Current amplitudes (mean ± SD) at +80 mV in the presence of different concentrations of VU0546110, B8, B9, and TL6-49 relative to that under control conditions (set to 1) (*n* ≥ 3). Continuous lines represent fit of the Hill equation yielding the dose–response relationships. (G) Dose–response relation of maximal Ca^2+^-signal amplitudes evoked by VU0546110, B8, B9, and TL6-49 in human sperm incubated in HTF relative to that evoked by progesterone (3 μM) (set to 1). (H) Ca^2+^-signal amplitude at 10-fold IC_50_-concentrations of VU0546110, B8, B9, and TL6-49 in human sperm incubated in HTF relative to that evoked by progesterone (3 μM), set to 1 (*n* = 3–5).

We conclude that VU0546110 (**1**), B8 (**4**), B9 (**26**), and TL6-49 (**32**) inhibit Slo3 with similar potency, i.e., the compounds feature low single-digit micromolar IC_50_ values ranging from 1.6 μM (TL6–49) to 4.8 μM (B8) (Figure 8F, Table S1). Moreover, B9 (**26**), but not B8 (**4**) and TL6-49 (**32**), is less potent than VU0546110 (**1**) in activating CatSper (Figure 8G). In addition, B8 (**4**), B9 (**26**) and TL6-49 (**32**) activate CatSper in human sperm with lower efficacy (Figure 8G). As a consequence, particularly B9 (**26**), but in fact also B8 (**4**) and TL6-49 (**32**), feature an enhanced selectivity window for Slo3 over CatSper than VU0546110 (**1**) (Figure 8H): for example, at a concentration of ten times the IC_50_ to inhibit Slo3 (i.e., 26 μM), VU0546110 (**1**) evokes Ca^2+^ signals with a signal amplitude of 0.53 ± 0.08 (*n* = 4) relative to that evoked by progesterone (set to 1). Under the same condition, the relative B9-evoked signal amplitude is only 0.19 ± 0.03 (*n* = 5). Therefore, we propose B9 (**26**) as a novel, improved tool compound to investigate the physiology of Slo3 in human sperm.

## Discussion

VU0546110 was introduced as the first selective pharmacological tool to elucidate the molecular and cellular physiology of Slo3 in human sperm.^40^ Here, we demonstrate that VU0546110 and its derivatives exhibit dual activity on both Slo3 and CatSper channels in human sperm. This finding challenges concepts regarding the function of Slo3 in human sperm that are based on the action of VU0546110.^40,54^ VU0546110 was also proposed as a good starting point to further advance the development of novel nonhormonal contraceptives that disable the function of sperm through inhibition of Slo3. Both Slo3 and CatSper represent promising targets for contraceptives as they are essential for human sperm function and fertilization.^16–18,44,45^ Thus, inhibiting either of these channels should prevent sperm to fertilize the egg and, thereby, prevent pregnancy. Moreover, as the channels are expressed exclusively in sperm, selective inhibitors may have minimal or no side effects. Yet, whereas molecules that affect both Slo3 and CatSper might have an even higher contraceptive potency, it is challenging to develop such multitarget compounds into safe drugs. Thus, not only to elucidate the role of Slo3 in human sperm, but also to advance Slo3 as a target for contraceptives, compounds that are selective for Slo3 over CatSper are needed.

The finding that VU0546110 also acts on CatSper is indeed not surprising. The pharmacological profiles of human CatSper and Slo3 are known to overlap substantially.^20,21,26,39,46–51^ Moreover, a plethora of studies has demonstrated that CatSper is promiscuously modulated by a wide range of ligands and synthetic compounds, including endocrine-disrupting chemicals (EDCs),^22,23,30^ odorants,^50^ pharmaceutical compounds,^8,22–24,30,32,55–57^ cannabinoids,^58^ and steroids.^33,60^ This requires scrutinizing whether compounds designed to affect a particular protein in human sperm may exhibit an off-target action on CatSper. Along this line: a new generation of Slo3 inhibitors, e.g., VU6032735, with enhanced potency to inhibit Slo3 and improved selectivity for Slo3 over hERG has already been introduced.^61^ Their action on CatSper remains to be elucidated before using them as pharmacological tools. Here, we identified two compounds, i.e., B8 (**4**), B9 (**26**), that inhibit Slo3 with similar potency as VU0546110, but feature a reduced potency and efficacy to activate CatSper. To date, among the compounds characterized for their action on both channels, B9 (**26**) represents the one with the highest selectivity for Slo3 over CatSper. This compound represents a promising new lead and may come into focus of more extensive structure–activity and –selectivity analysis in the future. This should also involve the analysis of the action of the two enantiomers.

One roadblock toward the design of selective inhibitors has been the lack of high-resolution structures of human CatSper and Slo3, elucidating their inhibitor-binding sites and the molecular underpinnings of inhibitor-protein interactions. This is required for structure-based drug design, which facilitates the development of potent and highly selective compounds. As long as high-resolution structures are lacking, the design and identification of novel, selective Slo3 inhibitors relies on traditional medicinal-chemistry driven design-make-test-analyze cycles and/or drug-screening campaigns. For the latter, we introduced a medium-throughput Ca^2+^-fluorometric assay that allows to simultaneously assess the agonistic and inhibitory action of compounds on CatSper and Slo3, respectively. Determining compound-evoked Ca^2+^ signals in control HTF versus K-HTF represents a time-effective approach to screen for Slo3 inhibitors that are selective for Slo3 over CatSper, minimizing laborious electrophysiological experiments. Even though this approach led to some false-positive hits, no false-negative results were observed, highlighting its suitability. Implementing this assay into the drug-screening strategy could ease the future identification of selective Slo3 inhibitors.

## Material and Methods

### Synthesis

Unless otherwise noted, moisture-sensitive reactions were conducted under dry nitrogen. Thin layer chromatography (tlc): Silica gel 60 F_254_ plates (Merck). Flash chromatography (fc): Silica gel 60, 40–64 μm (Merck); parentheses include: eluent, *R*_f_ value. Melting point: Melting point apparatus MP50 (Mettler Toledo), uncorrected. MS: MicroTOFQII (Bruker Daltonics): APCI.^1^H NMR (400 MHz), ^13^C NMR (100 MHz): Agilent DD2 400 and 600 MHz spectrometers.; δ in ppm related to tetramethylsilane; coupling constants are given with 0.5 Hz resolution; the assignments of ^13^C and ^1^H NMR signals were supported by 2D NMR techniques. IR: FT/IR IRAffinity-1 IR spectrometer (Shimadzu) using ATR technique.

### Quantitative NMR

Purity of the compound was analyzed with quantitative NMR spectroscopy as described by Pauli et al.^62^ In brief, 6–10 mg of the analyte were mixed with equimolar amounts of 1,3,5-trimethoxybenzene as internal calibrant (purity: 99.96%) and dissolved in CDCl_3_. Quantitative NMR spectra were recorded on an Agilent DD2 600 MHz NMR spectrometer. The 25 °C spectra were processed by ’Zero Filling’ to 256 K, apodization as stanning, manual phase correction and polynomial baseline correction (order 5). The purest signals were integrated, the peak area reported by intensity and the purity was calculated as described by Pauli et al.^62^

### Synthetic Procedures

#### 1-(Azepan-1-yl)-2-(4-fluorophenyl)­ethanone (**33**, TL6-47)

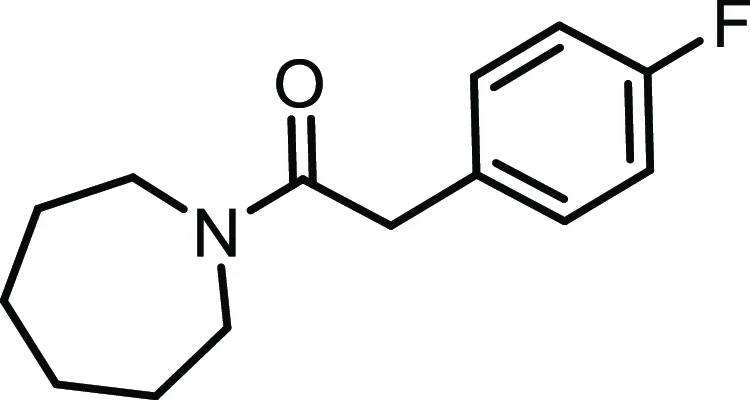



4-Fluorophenylacetyl chloride (249 mg, 1.4 mmol, 1.0 equiv), was added to a solution of azepane (439 mg, 3.1 mmol, 3.1 equiv) and DMAP (182 mg, 1.5 mmol, 1.0 equiv) in dry CH_2_Cl_2_ (10 mL) at 0 °C. After stirring for 15 min at 0 °C, the reaction mixture was stirred for 1 h at room temperature. Then, the reaction mixture was washed with 1 M HCl (10 mL) and brine (10 mL). The organic layer was separated, dried (Na_2_SO_4_), filtered and concentrated under reduced pressure. The crude product was purified by flash chromatography (cyclohexane/ethyl acetate 90:10 to 20:80).

Colorless solid, mp 51 °C, yield 299 mg (88%). C_14_H_18_FNO (235.3 g/mol).

**TLC**: Rf = 0.52 (cyclohexane/ethyl acetate 50/50).

**HR-MS** (APCI): *m*/*z* = 236.1442 (calcd. 236.1445 for C_14_H_19_FNO [M + H]^+^).

^**1**^ **H NMR** (400 MHz, CDCl_3_) δ (ppm) = 1.52 (m, 4H, 4-H_2 azepane_, 5-H_2 azepane_), 1.64 (m, 2H, 3-H_2 azepane_), 1.71 (m, 2H, 6-H_2 azepane_), 3.43 (m, 2H, 2-H_2 azepane_), 3.53 (m, 2H, 7-H_2 azepane_), 3.67 (d, *J* = 0.6 Hz, 2H, benzyl CH_2_), 7.00 (t, *J* = 8.7 Hz, 2H, 3,5-H_benzyl_), 7.23 (m, 2H, 2,6-H_benzyl_).

^**13**^ **C NMR** (151 MHz, CDCl_3_) δ (ppm) = 26.8 (C-4_azepane_), 27.3 (C-5_azepane_), 27.6 (C-6_azepane_), 29.3 (C-3_azepane_), 40.2 ((C=O)­NH–*C*H), 46.3 (C-7_azepane_), 48.4 (C-2_azepane_), 115.6 (d, *J* = 21.6 Hz, C-3_benzyl_, C-5_benzyl_), 130.5 (d, *J* = 8.3 Hz, C-2_benzyl_, C-6_benzyl_), 131.3 (d, *J* = 3.5 Hz, C-1_benzyl_), 161.9 (d, *J* = 245 Hz, C-4_benzyl_), 170.6 ((*C*=O)­NH–CH).

**FT-IR**: ν̃ [cm^–1^] = 2913 (C–H_aliph._); 2855 (C–H_aliph._); 1628 (C=O); 1412 (CN_amide_); 791 (Ar–H_out of plane_).

**q-NMR**: 96.2% (IC = TMB).

#### 1-(3-(4-Chlorophenyl)­azepan-1-yl)-2-(4-fluorophenyl)­ethanone (**32**, TL6-49)

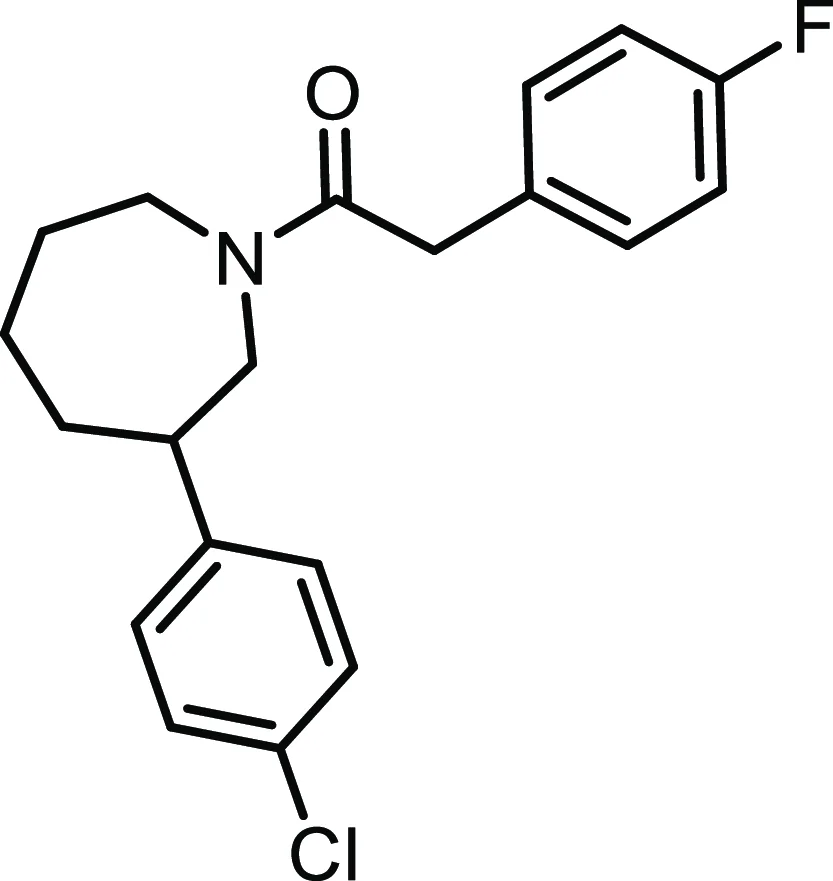



4-Fluorophenylacetyl chloride (124 mg, 0.72 mmol, 1.7 equiv) was added to a solution of 3-(4-chlorophenyl)­azepane (103 mg, 0.42 mmol, 1.0 equiv) and DMAP (142 mg, 1.2 mmol, 2.8 equiv) in dry CH_2_Cl_2_ (10 mL) at 0 °C. After stirring for 5 min at 0 °C, the reaction mixture was stirred for 1.5 h at room temperature. Then, the reaction mixture was washed with 1 M HCl (10 mL) and brine (10 mL). The organic layer was separated, dried (Na_2_SO_4_), filtered and concentrated under reduced pressure. The crude product was purified by reversed phase flash chromatography (acetonitrile/H_2_O 10:90 to 100:0).

Colorless oil, yield 30 mg (21%). C_20_H_21_ClFNO (345.8 g/mol).

**TLC**: Rf = 0.31 (CH_2_Cl_2_/methanol 90/10 + 1% dimethylethylamine).

**HR-MS** (APCI): *m*/*z* = 346.1369 (calcd. 346.1368 for C_20_H_22_ClFNO [M + H]^+^).

#### Major Rotamer (58%)

^**1**^ **H NMR** (400 MHz, CDCl_3_) δ (ppm) = 1.23–1.46 (m, 1H, 5-H_azepane_), 1.51–1.77 (m, 2H, 4-H_azepane_, 6-H_azepane_), 1.80–1.99 (m, 2H, 4-H_azepane_, 5-H_azepane_), 1.99–2.12 (m, 1H, 6-H_azepane_), 2.86–3.00 (m, 2H, 2-H_azepane_, 3-H_azepane_), 3.27–3.45 (m, 1H, 7-H_azepane_), 3.64–3.80 (m, 3H, 7-H_azepane_, CH_2 benzyl_), 4.15–4.29 (m, 1H, 2-H_azepane_), 6.93–7.08 (m, 2H, 3,5-H_benzyl_), 7.14–7.35 (m, 6H, 2,6-H_benzyl_, 2,6-H_phenyl_, 3,5-H_phenyl_).

^**13**^ **C NMR** (101 MHz, CDCl_3_): δ (ppm) = 25.2 (C-5_azepane_), 26.9 (C-6_azepane_), 35.0 (C-4_azepane_), 40.1 ((C=O)­NH–*C*H), 44.7 (C-3_azepane_), 48.3 (C-7_azepane_), 52.9 (C-2_azepane_), 115.5 (d, *J* = 21.4 Hz, C-3_benzyl_, C-5_benzyl_), 128.3–129.0 (C-2_phenyl_, C-3_phenyl_, C-5_phenyl_, C-6_phenyl_), 130.6 (d, *J* = 8.0 Hz, C-2_benzyl_, C-6_benzyl_), 131.2 (d, *J* = 3.4 Hz, C-1_benzyl_), 132.2 (C-4_phenyl_), 143.0 (C-1_phenyl_), 162.0 (d, *J* = 242 Hz, C-4_benzyl_), 170.7 ((*C*=O)­NH–CH).

#### Minor Rotamer (42%)

^**1**^ **H NMR** (400 MHz, CDCl_3_) δ (ppm) = 1.23–1.46 (m, 1H, 5-H_azepane_), 1.51–1.77 (m, 2H, 4-H_azepane_, 6-H_azepane_), 1.80–1.99 (m, 3H, 4-H_azepane_, 5-H_azepane_, 6-H_azepane_), 2.59 (tt, *J* = 11.0, 3.8 Hz, 1H, 3-H_azepane_), 3.15–3.27 (m, 1H, 2-H_azepane_), 3.27–3.45 (m, 1H, 7-H_azepane_), 3.64–3.80 (m, 3H, 2-H_azepane_, benzyl CH_2_), 3.87–3.99 (m, 1H, 7-H_azepane_), 6.93–7.08 (m, 4H, 3,5-H_benzyl_, 2,6-H_phenyl_), 7.14–7.35 (m, 4H, 2,6-H_benzyl_, 3,5-H_phenyl_).

^**13**^ **C NMR** (101 MHz, CDCl_3_): δ (ppm) = 26.1 (C-5_azepane_), 28.6 (C-6_azepane_), 34.8 (C-4_azepane_), 40.6 ((C=O)­NH–*C*H), 46.3 (C-7_azepane_), 47.7 (C-3_azepane_), 55.1 (C-2_azepane_), 115.8 (d, *J* = 21.1 Hz, C-3_benzyl_, C-5_benzyl_), 128.3–129.0 (C-2_phenyl_, C-3_phenyl_, C-5_phenyl_, C-6_phenyl_), 130.4 (d, *J* = 8.4 Hz, C-2_benzyl_, C-6_benzyl_), 131.2 (d, *J* = 3.4 Hz, C-1_benzyl_), 132.7 (C-4_phenyl_), 142.7 (C-1_phenyl_), 162.0 (d, *J* = 242 Hz, C-4_benzyl_), 170.7 ((*C*=O)­NH–CH).

**FT-IR**: ν̃ [cm^–1^] = 2924 (C–H_aliph._); 1632 (C=O); 1427 (CN_amide_); 822 (Ar–H_out of plane_), 795 (Ar–H_out of plane_).

**q-NMR**: 98.2% (IC = TMB).

**32** (TL6-49) represents the racemic mixture.

### Sperm Preparation

Semen samples were provided by normozoospermic donors as well as an infertile patient with a homozygous deletion of the *CATSPER*2 gene (for details see^17,52^ ) with prior written consent, according to the protocols approved by the Ethics Committee of the Ärztekammer Westfalen-Lippe and the Medical Faculty Münster (2021-402-f-S) and the Declaration of Helsinki. Motile sperm used for Ca^2+^ fluorometric experiments were purified by the swim-up procedure as described before.^17^ In brief, in 50 mL falcon tubes angled at 45°, 4 mL of human tubular fluid (HTF) medium, containing (in mM) 93.8 NaCl, 4.69 KCl, 0.2 MgSO_4_, 0.37 KH_2_PO_4_, 2.04 CaCl_2_, 0.33 Na-pyruvate, 21.4 lactic acid, 2.78 glucose, 21 HEPES, 4 NaHCO_3_ (pH adjusted to 7.35 with NaOH) was underlaid with 0.7 to 1.2 mL of ejaculate. After incubation for 1 h at 37 °C, supernatants were collected, and sperm were washed twice with HTF by centrifugation at 700 × g for 20 min. After the second centrifugation, sperm were resuspended to 10^7^ cells/mL in HTF containing 3 mg/mL human serum albumin (HSA, Irvine Scientific) and incubated at 37 °C. For comparison of compound action in absence and presence of HSA, sperm were resuspended to 10^7^ cells/m in HTF without HSA.

Alternatively, the liquefied semen was diluted 1:10 in HTF and sperm, somatic cells, and cell debris were pelleted by centrifugation (700 × g, 20 min, 37 °C). The sediment was then resuspended in the same volume of HTF. Aliquots of this suspension were pelleted using a centrifuge with a fixed-angle rotor (700 × g, 5 min, room temperature) and motile sperm were allowed to swim out. Washing and density adjustment were performed as described above.

### Measurement of Changes of the Intracellular Ca^2+^ Concentration

Changes of [Ca^2+^]_i_ in motile human sperm were measured in population using a plate reader (Fluostar Omega, BMG Labtech) in 384-well plates and fluorescence readout as described previously with minor modifications.^17,23^ Sperm were loaded with the fluorescent Ca^2+^ indicator Fluo-4 by incubation with Fluo-4-AM (5 μM, ThermoFisher) in the presence of Pluronic F-127 (0.05% w/v) for 20 min. Centrifugation at 700 × g for 5 min and resuspension to 1.75 × 10^7^ cells/mL in HTF was done to remove excess dye. For experiments in K-HTF, sperm were resuspended in K-HTF containing (in mM) 98.5 KCl, 0.2 MgSO_4_, 0.37 KH_2_PO_4_, 2.04 CaCl_2_, 21.4 lactic acid, 2.78 glucose, 21 HEPES, 4 KHCO_3_ (pH adjusted to 7.35 with KOH). For the measurements, wells were filled with 30 μL of the suspension of dye-loaded sperm. Fluorescence was excited at 480 nm and emission was recorded at 520 nm. After recording a baseline, 30 μL of the respective compound solution was added and the change in fluorescence was recorded. In parallel, a progesterone solution was added in one well to serve as control and reference for compound evoked Ca^2+^ signals. In experiments with *CATSPER*2^–/–^ sperm, the Ca^2+^ ionophore ionomycin was applied as a control and reference. Therefore, ionomycin was added 8 min after the addition of the compounds. Solutions were added using an electric multichannel Eppendorf pipette. Changes in Fluo-4 fluorescence, i.e., [Ca^2+^]_i_, are depicted as Δ*F*/*F* (%), which means the change in fluorescence (Δ*F*) relative to the mean basal fluorescence (*F*) before application of buffer or compounds/ligands to correct for intra- and interexperimental variations in basal fluorescence among individual wells or to correct for compound-evoked changes in fluorescence (see below). For examination of compound-evoked Ca^2+^ signal, basal fluorescence (*F*) was determined before addition of compounds. To ease comparisons between experiments, Δ*F*/*F* was normalized to the maximal signal amplitude evoked by progesterone. EC_50_ values were calculated by determining the normalized maximal signal amplitudes evoked by the ligand in the presence of various compound concentrations. Data from fluorescence recordings was analyzed in GraphPad Prism version 10.4.1.

### Electrophysiological Recordings of Slo3 Currents

CHO cells (RRID: CVCL_0214) were cotransfected as described before^46^ with a pcDNA3.1­(+) vector containing the full-length coding sequence of human Slo3 (NM_001031836) modified with a carboxy-terminal hemagglutinin tag (HA-tag) and in which the sequence coding for the neomycin resistance gene was replaced by the coding sequence for citrine, and a pcDNA3.1­(+) vector containing a sequence encoding hLRRC52-mCherry (NM_001005214). Cells were cotransfected with 2–3 ng/μL of the respective vector for 6–8 h using Lipofectamine 2000 (Invitrogen) in Opti-MEM (Gibco). Afterward, cells were cultivated in F-12 medium (F-12 Nut Mix + GlutaMAX (Gibco)) with 10% FBS, 2 mM l-glutamine, and 1% Anti-Anti (Thermo Fisher) at 37 °C and 5% CO_2_; after another 24 h, cells were transferred onto poly-L-lysine-coated cover slides with a density of 15.000 cells per well of a four-well plate and stimulated with 5 mM Na-Butyrate at least 12 h before the experiment.

Electrophysiological recordings were performed in the whole-cell configuration as described before^39^ using an Axopatch 200B patch clamp amplifier (Molecular Devices, Sunnyvale, CA, USA) controlled by Clampex 10.7 (Molecular Devices). Signals were low-pass filtered at 10 kHz with a four-pole Bessel filter and digitized with a Digidata 1440A data acquisition system (Molecular Devices). Series resistance and cell capacitance were compensated. A step protocol with steps from −100 mV to +150 mV followed by a step to +50 mV from a holding potential of −80 mV was used. The intracellular solution contained 130 mM K-Aspartate, 10 mM NaCl, 1 mM EGTA, 20 mM HEPES, pH 7.3 with KOH and the pipet resistance was 4–6 MΩ. Cells were perfused with extracellular solution (140 mM NaCl, 5.4 mM KCl, 1 mM MgCl_2_, 1.8 mM CaCl_2_, 5 mM Hepes, 10 mM Glucose, pH 7.4 with NaOH). All substances were diluted in extracellular solution and applied to the cells by a gravity-driven perfusion system. Experiments were performed at room temperature.

Statistical analysis of data was done using OriginPro (Origin 2022b; OriginLab Corporation, Northampton, MA, USA). Calculation of IC_50_ values was done in Origin using the Hill1 equation $y=\frac{x^{n}}{k^{n}+x^{n}}$ . Plotting of data was done using Origin and GraphPad Prism version 10.4.1 (Prism, La Jolla, United States).

### Patch-Clamp Recordings from Human Sperm

CatSper currents from human sperm were recorded in the whole-cell configuration as described before.^20^ We formed the seals between the sperm and the pipet (10–15 MΩ) at the cytoplasmic droplet in the neck region. The pipette solution (IS) contained (in mM) 130 Cs-aspartate, 50 HEPES, 5 EGTA, 5 CsCl adjusted to pH 7.4 using CsOH. Seals were formed in extracellular solution (HS) containing (in mM) 140 NaCl, 5 KCl, 1 MgSO_4_, 2 CaCl_2_, 20 HEPES, and 5 Glucose, adjusted to pH 7.45 using NaOH. We recorded monovalent currents in a sodium-based divalent-free solution (NaDVF) containing (in mM) 140 mM NaCl, 40 HEPES, and 1 EGTA. Data were not corrected for liquid junction potential.

## Supplementary Material



## Notes

Figures S1–S5: Additional data on the action of VU0546110 and derived compounds on CatSper and CatSper-mediated Ca^2+^ signals in human sperm. Table S1: Summary of the pharmacological profile of VU0546110 and derived compounds ^1^H and ^13^C NMR spectra of **32** and **33** (PDF)
