# Supplementary material for: Off-Target Activation of CatSper Limits the Utility of a Novel Small Molecule Slo3 Inhibitor and its Derivatives in Human Sperm
Source: ACS Pharmacol Transl Sci. 2026 Aug 31;9(9):2469–81. doi: 10.1021/acsptsci.6c00091 (PMC13632778; doi:10.1021/acsptsci.6c00091)

Supporting Information

Off-target activation of CatSper limits the utility of a novel small molecule Slo3 inhibitor and its derivatives in human sperm

Teresa Mittermair[1,‖], Jens Münchow[2,‖], Timo Strünker[1*], Louisa Temme[3*], Christoph Brenker[1*]

[1]Centre of Reproductive Medicine and Andrology, University Hospital Münster, University of Münster, 48149 Münster, Germany, [2] Institute of Pharmaceutical and Medicinal Chemistry, Pharmacampus, University of Münster, 48149 Münster, Germany, [3]Institute of Pharmacy, University of Hamburg, 20148 Hamburg, Germany.

‖ shared first authors, * shared corresponding authors

Corresponding authors:

Timo Strünker: Tel.: +49-251-8358238; Email: timo.struenker@ukmuenster.de

Louisa Temme: Tel.: +49-40-2395-23441; Email: louisa.temme@uni-hamburg.de

Christoph Brenker: +49-251-8358240; Email: christoph.brenker@ukmuenster.de

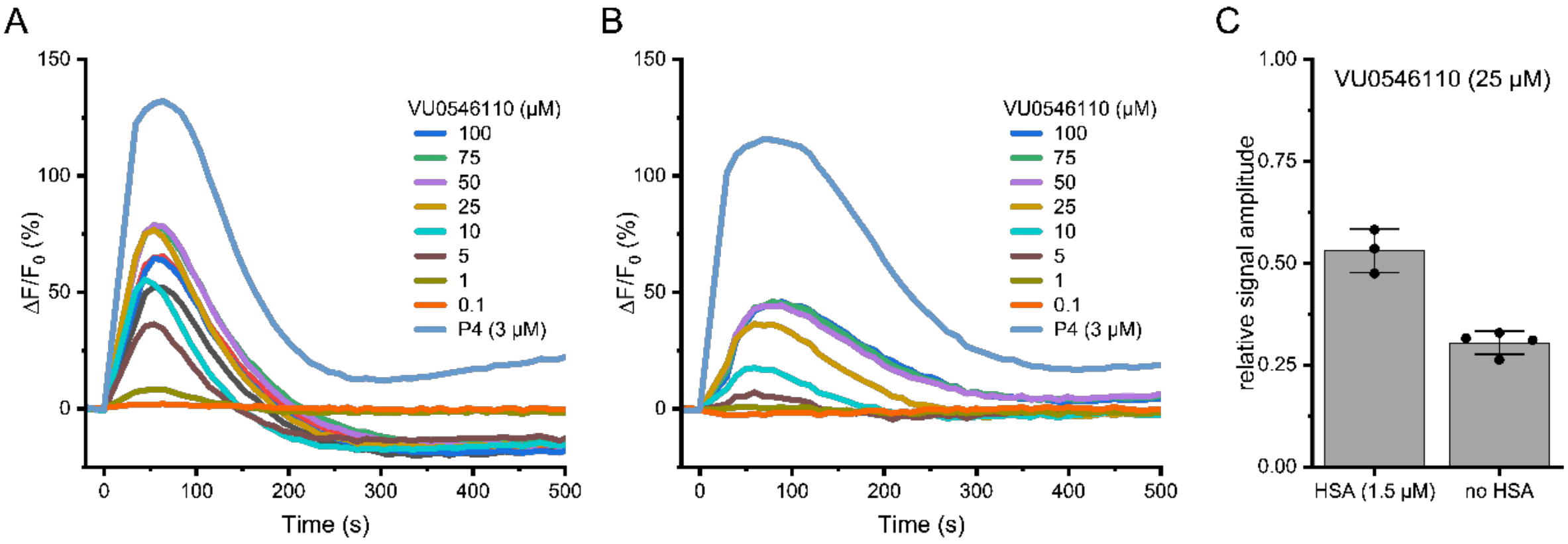

Supplementary Figure 1. Action of HSA on VU0546110-induced Ca^{2+} signals. A) Representative Ca^{2+} signals in human sperm evoked by different concentrations of VU0546110 (**1**) and progesterone (3 µM) in the presence of HSA (1.5 µM) in HTF. $\Delta F/F_0$ (%) indicates the percentage change in fluorescence (ΔF) with respect to the mean basal fluorescence (F_0) before the application of VU0546110 (**1**). B) Representative Ca^{2+} signals in human sperm evoked by different concentrations of VU0546110 (**1**) and progesterone (3 µM) in the absence of HSA in HTF. $\Delta F/F_0$ (%) indicates the percentage change in fluorescence (ΔF) with respect to the mean basal fluorescence (F_0) before the application of VU0546110 (**1**). C) Maximal amplitudes of Ca^{2+} signals in human sperm evoked by VU0546110 (**1**, 25 µM) in the presence (1.5 µM) and absence of HSA (mean ± SD, n = 3 and 4) relative to that evoked by progesterone (3 µM), set to 1.

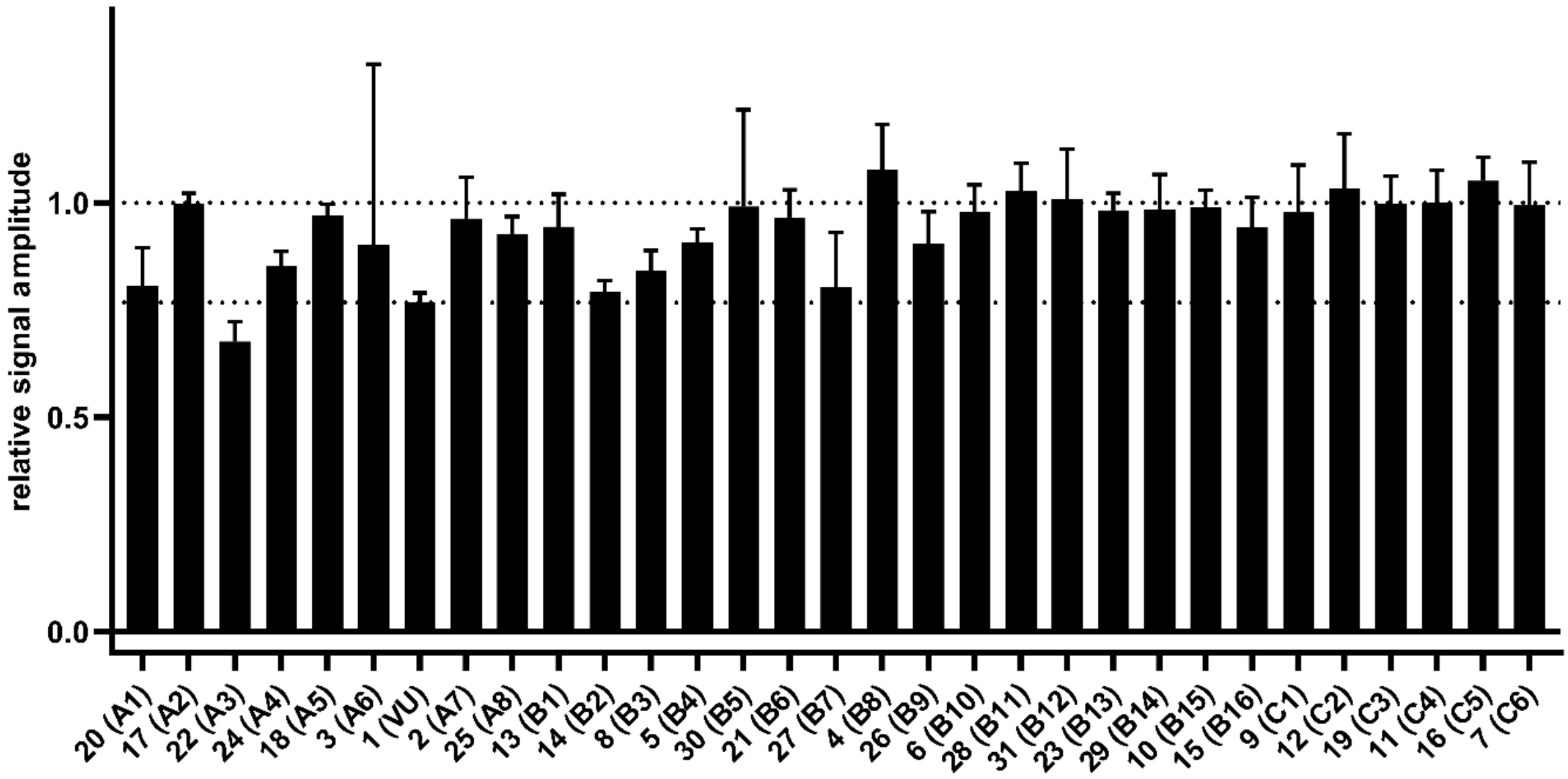

Supplementary Figure 2. Action of VU0546110 and derived compounds on progesterone-evoked Ca^{2+} signals in human sperm. Maximal (± SD) amplitude of progesterone (3 µM)-evoked Ca^{2+} signals in human sperm in the presence of indicated compounds (30 µM) in K-HTF, relative to that evoked by progesterone (3 µM) in the absence of any compound (set to 1). Number in brackets indicate the numbering and grouping of the compounds according to the amplitude of the compound-evoked Ca^{2+} responses in Classes A, B and C. See also Figure 3 and the explanation in the text.

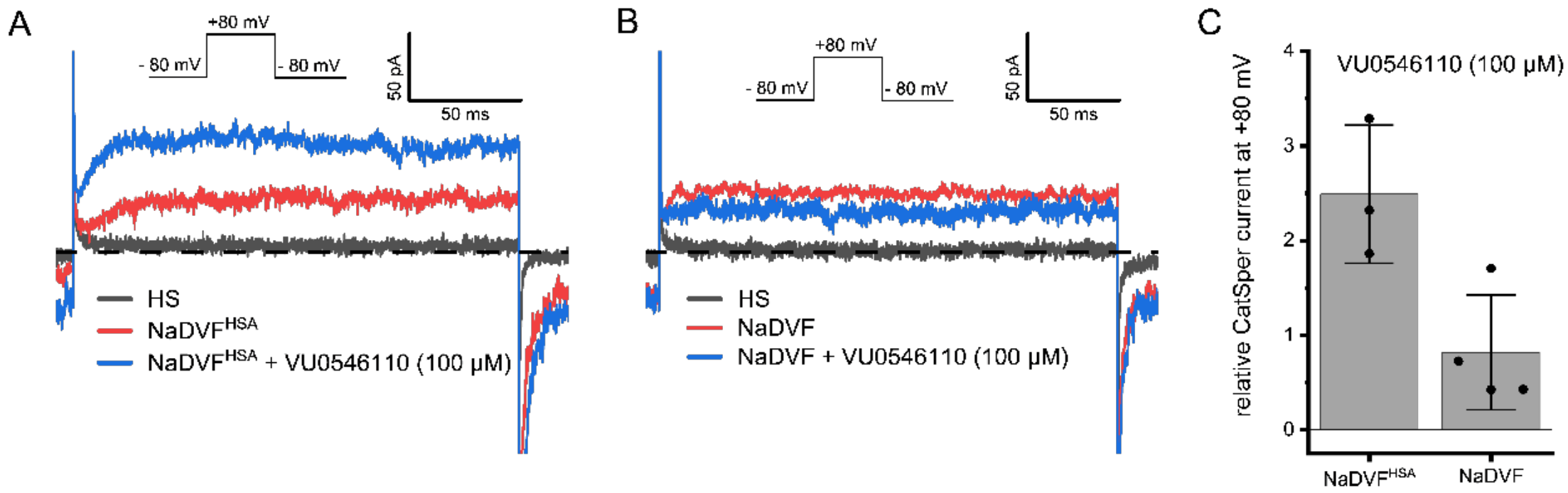

Supplementary Figure 3. Action of HSA on VU0546110-evoked increase of CatSper currents. A) Representative CatSper-mediated membrane currents recorded from a human sperm cell in the whole-cell configuration in extracellular solution containing Ca^{2+} and Mg^{2+} (HS), in divalent-free Na^{+}-based bath solution (NaDVF) containing HSA (15 µM, $NaDVF^{HSA}$), and after perfusion with $NaDVF^{HSA}$ containing VU0546110 (**1**, 100 µM) as shown in Figure 1G. B) Representative CatSper-mediated membrane currents recorded from a human sperm cell in the whole-cell configuration in HS, NaDVF, and after perfusion with NaDVF containing VU0546110 (**1**, 100 µM). C) Mean (± SD) CatSper currents at +80 mV in $NaDVF^{HSA}$ versus NaDVF both containing VU0546110 (**1**, 100 µM) (n = 3-4).

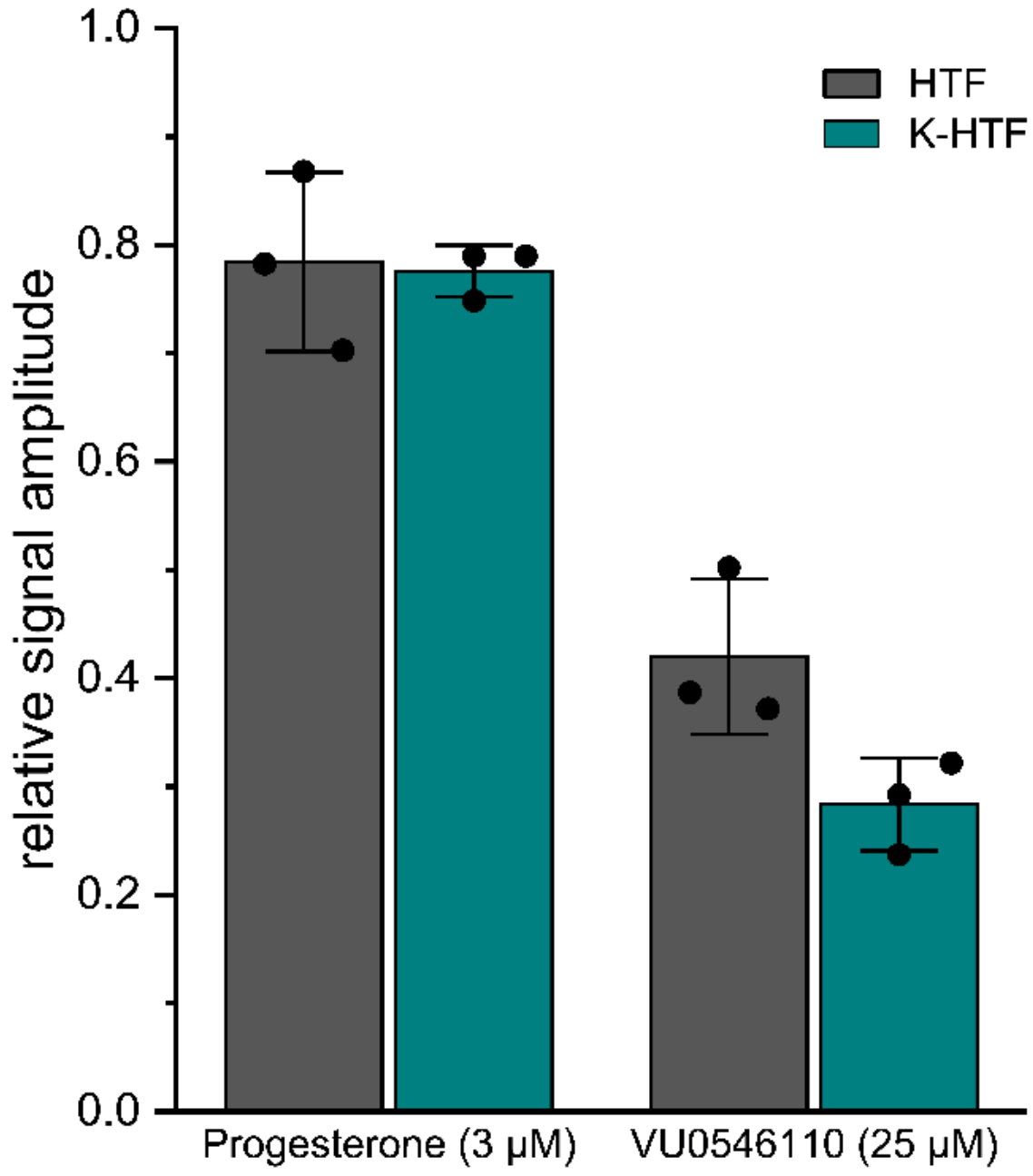

Supplementary Figure 4. Amplitudes of progesterone- and VU0546110-evoked Ca^{2+} signals in HTF and K-HTF. A) Mean (± SD) maximal Ca^{2+} signals evoked by progesterone (3 µM) and VU0546110 (**1**, 25 µM) in HTF and K-HTF relative to that evoked by ionomycin (10 µM) (set to 1) (n = 3).

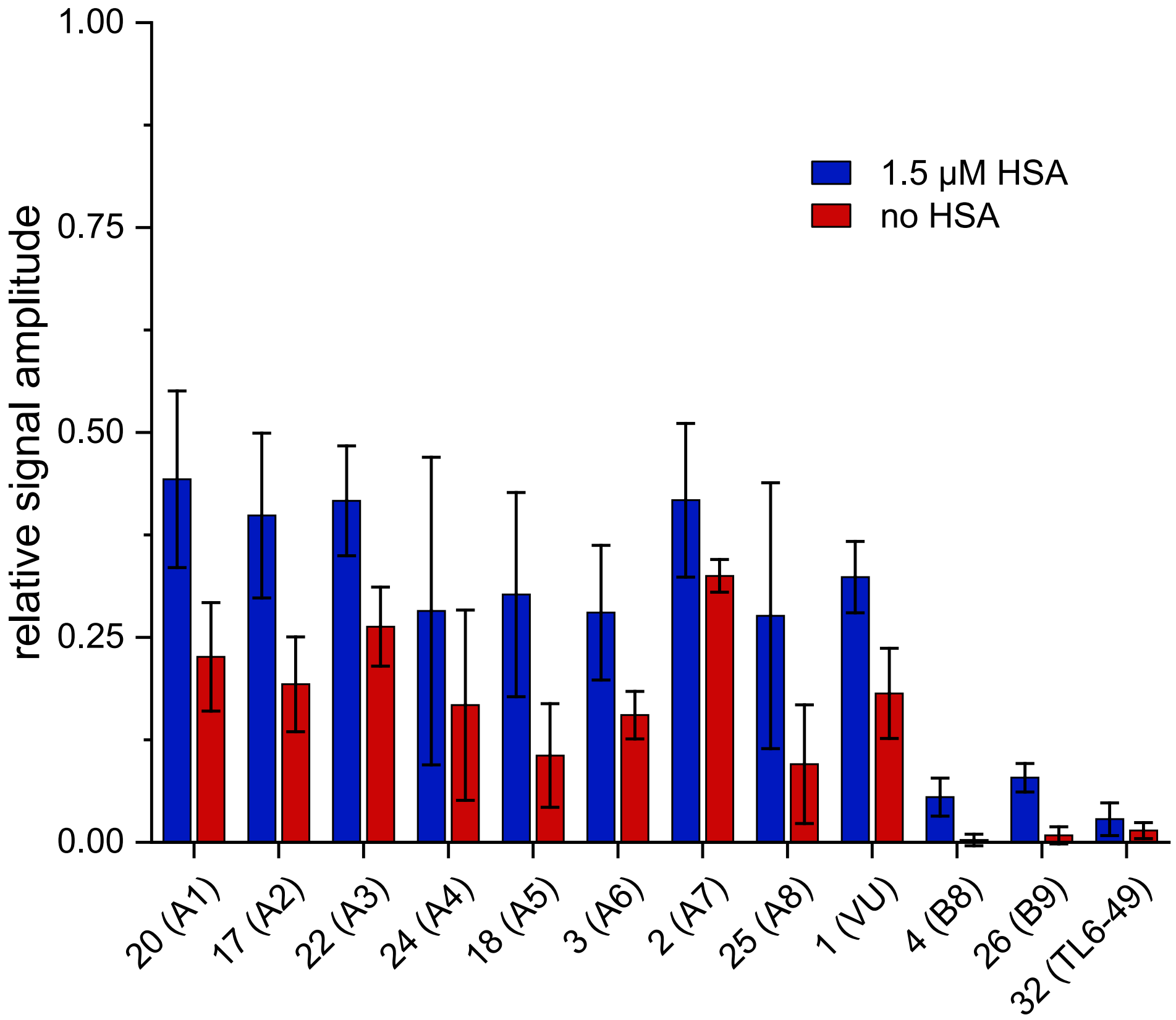

Supplementary Figure 5. Amplitude of Ca^{2+} signals evoked by VU0546110-derived compounds in K-HTF in the presence and absence of HSA. Blue: Maximal (± SD) amplitude of Ca^{2+} signals in human sperm evoked by compounds (30 µM) in the presence of HSA (1.5 µM) in K-HTF relative to that evoked by progesterone (3 µM) (set to 1) (n ≥ 3). Red: Maximal (± SD) amplitude of Ca^{2+} signals in human sperm evoked by compounds (30 µM) in absence of HSA in K-HTF relative to that evoked by progesterone (3 µM) (set to 1) (n ≥ 3).

	IC_{50} Slo3 (µM)	EC_{50} CatSper (µM)	Efficacy CatSper	Ca^{2+} signal amplitude at 10-fold IC_{50}
VU0546110 (**1**)	2.6 ± 0.4	12.6 ± 1.2	0.67 ± 0.12	0.53 ± 0.08
B8 (**4**)	4.8 ± 0.9	14.4 ± 2.4	0.37 ± 0.02	0.32 ± 0.03
B9 (**26**)	3.8 ± 0.3	28.0 ± 12.2	0.30 ± 0.05	0.19 ± 0.03
TL6-49 (**32**)	1.6 ± 0.5	5.8 ± 0.8	0.43 ± 0.05	0.38 ± 0.01

Supplementary Table1. Summary of the pharmacological profiles of VU0546110 and derivatives. Comparison of IC_{50} values for the inhibition of heterologously expressed Slo3 channels and EC_{50} values for stimulation of CatSper-mediated Ca^{2+} signals. The efficacy for CatSper activation is given relative to progesterone (set to 1).

NMR spectra

Compound TL6-49 (**32**):

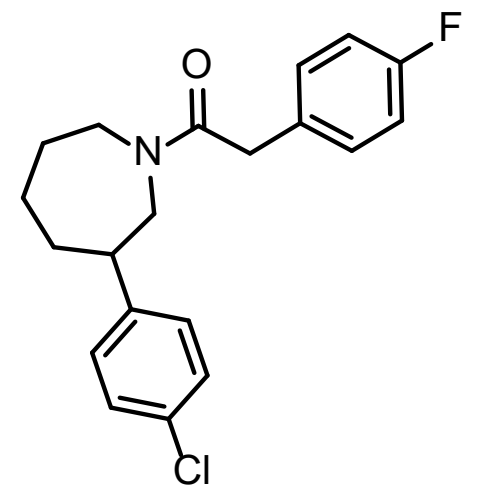

^{1}H NMR spectrum:

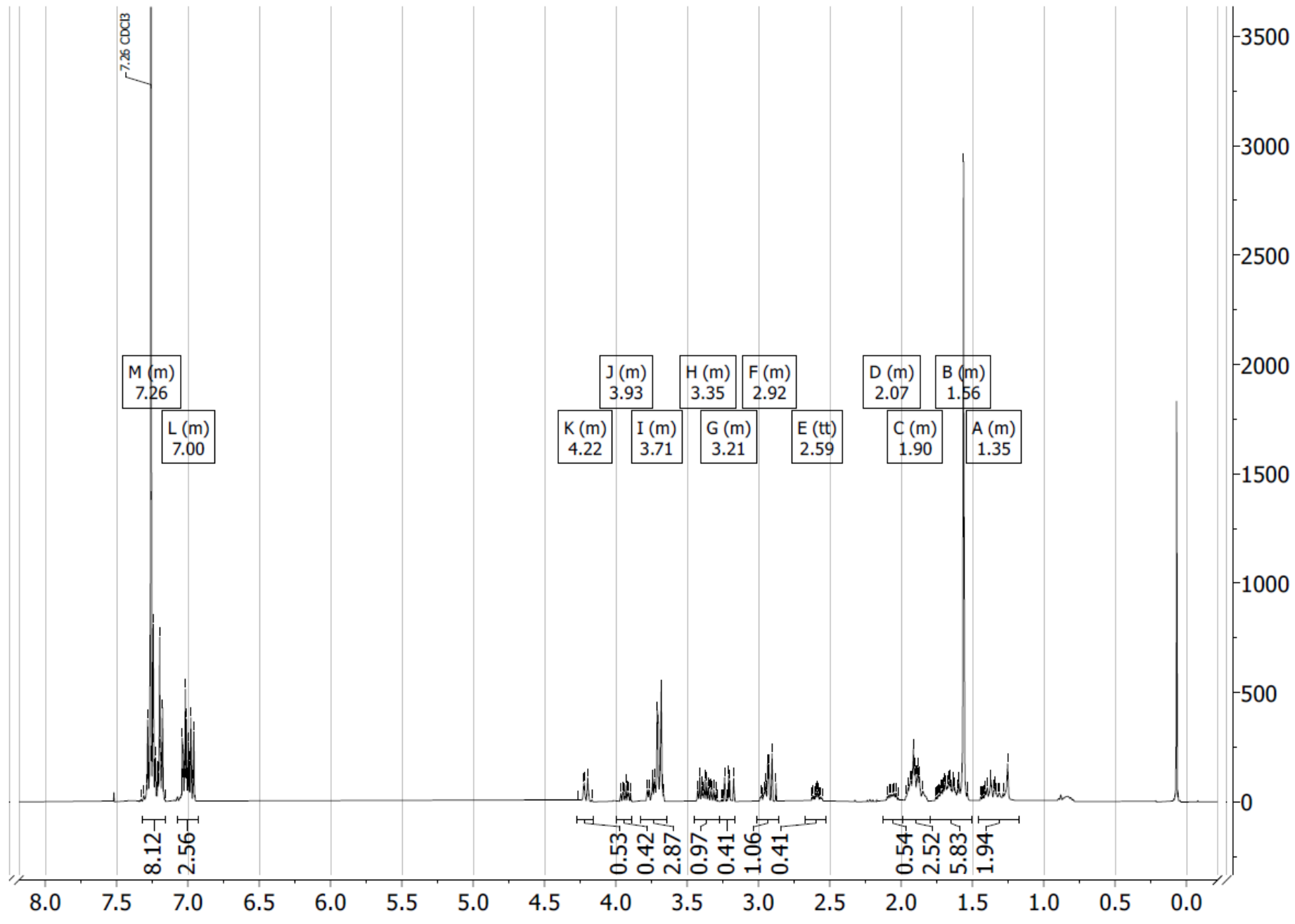

^{13}C NMR spectrum:

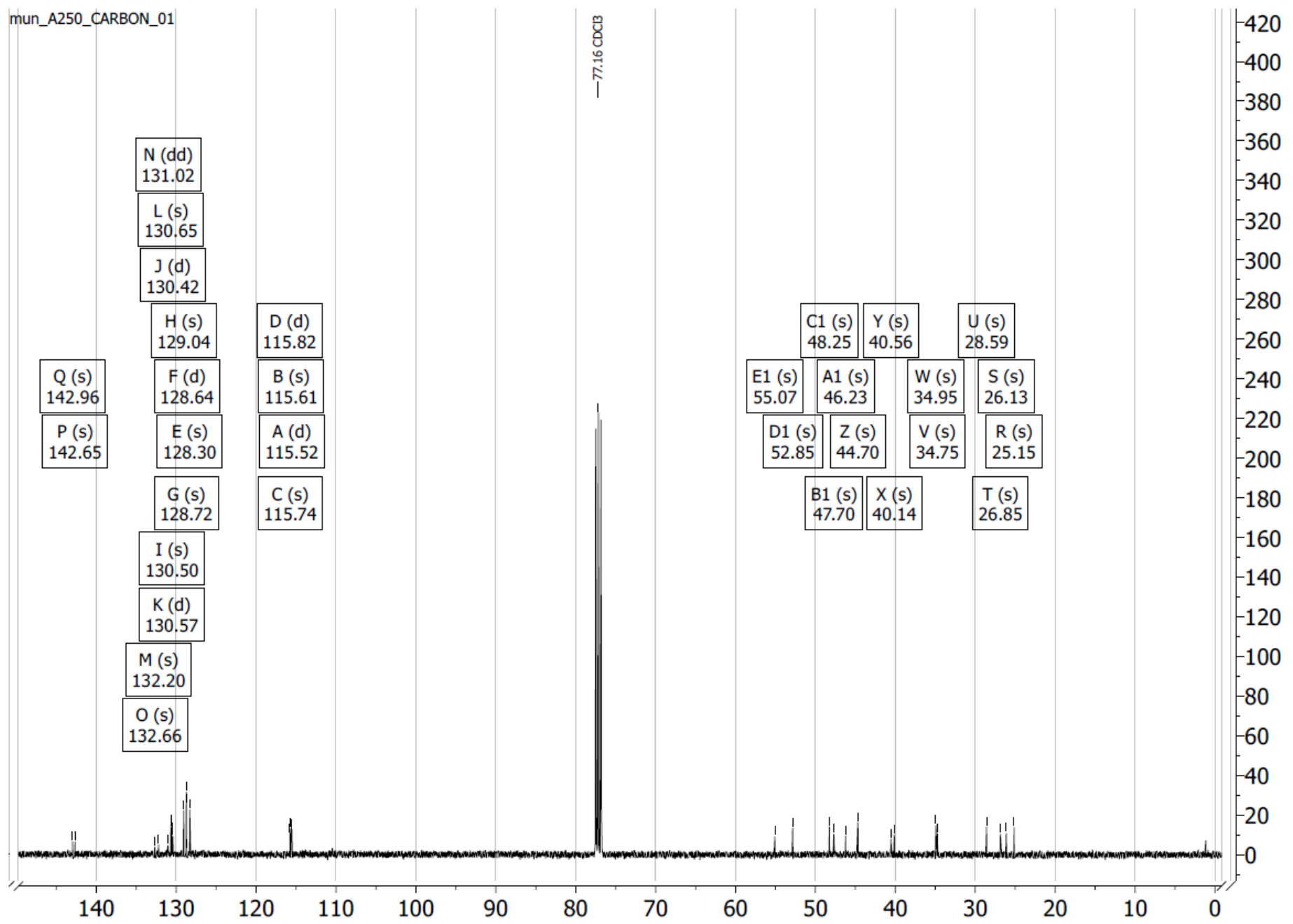

Compound TL6-47 (**33**):

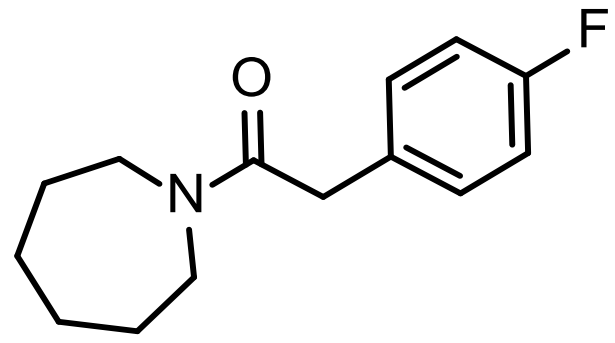

^{1}H NMR spectrum:

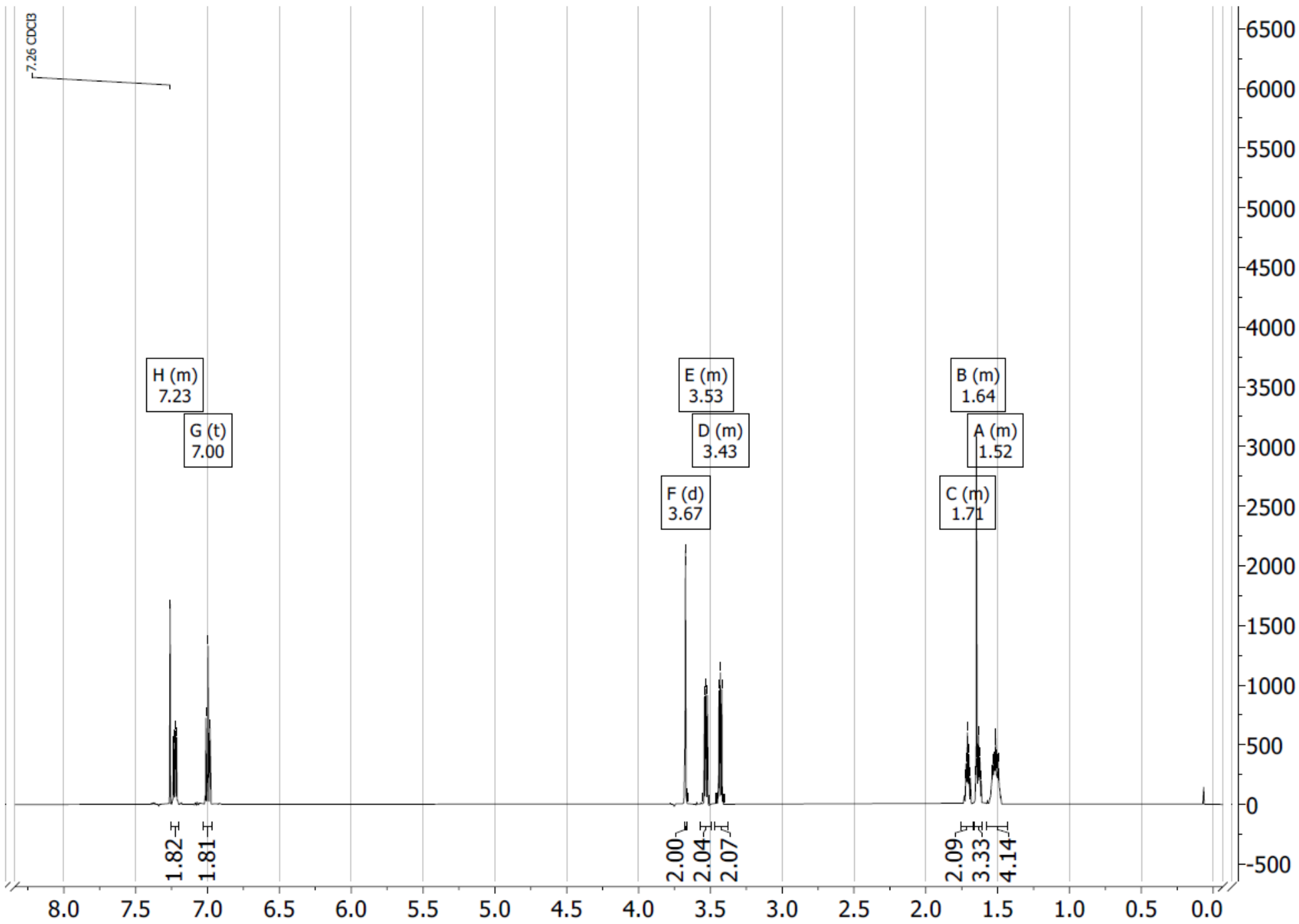

^{13}C NMR spectrum:

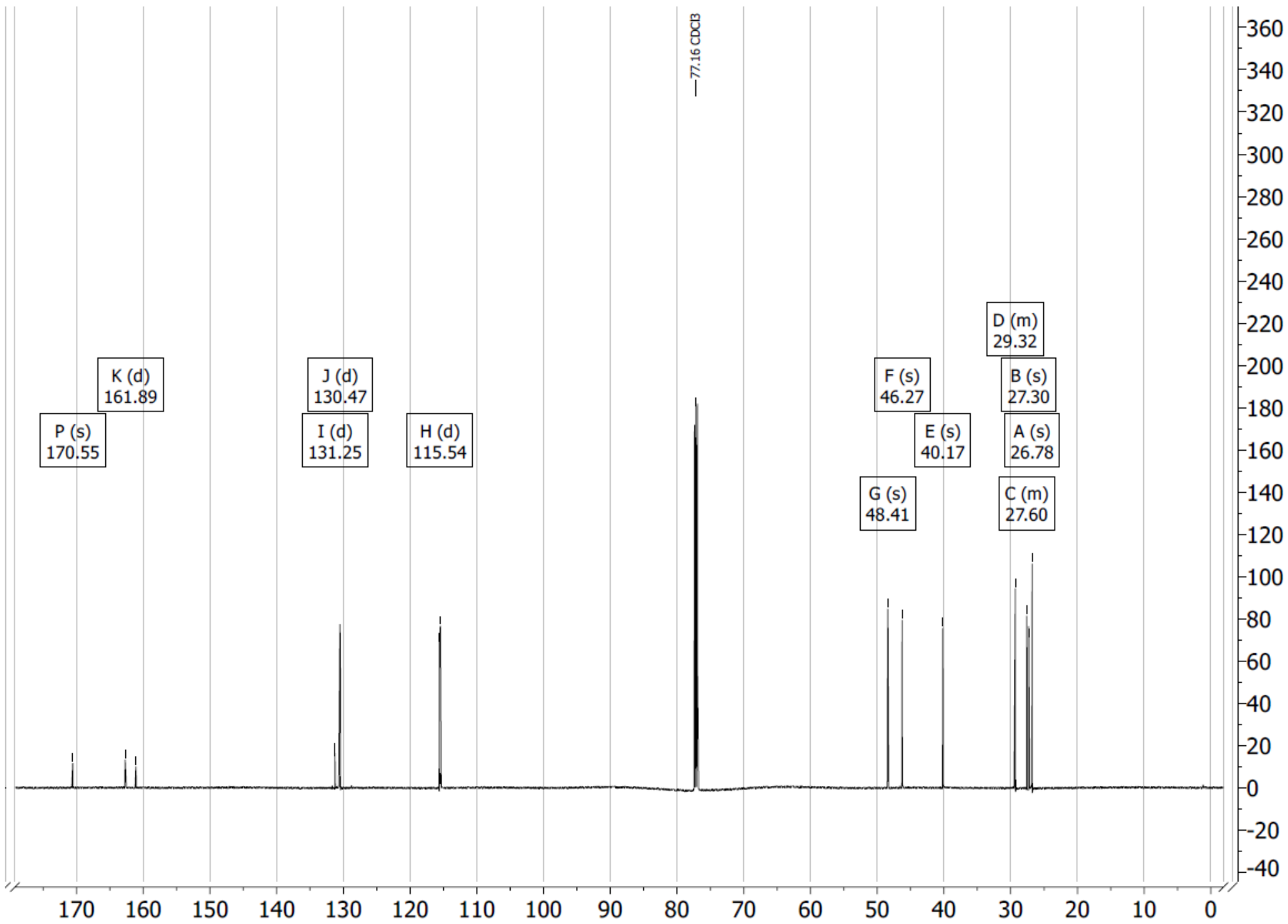

Supplement: Supplementary file 1 [file pt6c00091_si_001.pdf]
